# Fluorogenic Probes with Substitutions at the 2 and 7 Positions of Cephalosporin are Highly BlaC-Specific for Rapid *Mycobacterium tuberculosis* Detection**

**DOI:** 10.1002/anie.201405243

**Published:** 2014-07-02

**Authors:** Yunfeng Cheng, Hexin Xie, Preeti Sule, Hany Hassounah, Edward A Graviss, Ying Kong, Jeffrey D Cirillo, Jianghong Rao

**Affiliations:** Molecular Imaging Program at Stanford, Departments of Radiology and Chemistry, Stanford University1201 Welch Road, Stanford, CA 94305-5484 (USA); Department of Microbial Pathogenesis and ImmunologyTexas A&M Health Science Center (USA); Department of Pathology and Genomic MedicineHouston Methodist Research Institute (USA); Department of Microbiology, Immunology, and BiochemistryUniversity of Tennessee (USA)

**Keywords:** diagnostic tests, fluorogenic probes, lactams, β-lactamases, *Mycobacterium tuberculosi*s

## Abstract

Current methods for the detection of *Mycobacterium tuberculosis* (Mtb) are either time consuming or require expensive instruments and are thus are not suitable for point-of-care diagnosis. The design, synthesis, and evaluation of fluorogenic probes with high specificity for BlaC, a biomarker expressed by Mtb, are described. The fluorogenic probe CDG-3 is based on cephalosporin with substitutions at the 2 and 7 positions and it demonstrates over 120 000-fold selectivity for BlaC over TEM-1 Bla, the most common β-lactamase. CDG-3 can detect 10 colony-forming units of the attenuated *Mycobacterium bovis* strain BCG in human sputum in the presence of high levels of contaminating β-lactamases expressed by other clinically prevalent bacterial strains. In a trial with 50 clinical samples, CDG-3 detected tuberculosis with 90 % sensitivity and 73 % specificity relative to Mtb culture within one hour, thus demonstrating its potential as a low-cost point-of-care test for use in resource-limited areas.

Tuberculosis (TB) is a highly infectious airborne disease caused by the widely spread pathogen *Mycobacterium tuberculosis* (Mtb). It infects around one-third of the world’s population and claims the lives of 1.5 million people each year.[1] The worldwide emergence of multidrug-resistant tuberculosis (MDR-TB), extensively drug-resistant tuberculosis (XDR-TB), and totally drug-resistant tuberculosis (TDR-TB) further worsens this global health crisis.[1c,^2^] An important step in containing the spread and decreasing the mortality rate of this deadly airborne disease is rapid and timely detection of Mtb, preferably at the point-of-care (POC).[3] The extremely slow growth rate of the virulent Mtb pathogen, however, presents the largest hurdle to overcome. As a direct consequence, the gold standard culture-based technique and smear microscopy for TB diagnosis are limited to patients with advanced infection and usually take several weeks to produce a definitive diagnosis.[3c,d] Although nucleic acid based diagnostic methods such as Xpert provide sensitive and specific diagnosis,[4] the cost and requirement of highly skilled technical personnel and sophisticated instrument calibration make them less accessible in developing countries, where TB prevalence is highest.[5]

The successful development of a POC TB test depends on an Mtb-specific biomarker. Besides nucleic acids, lipoarabinomannan (LAM)[6] and unnatural trehalose analogues[7] were recently used as Mtb signatures for Mtb imaging and detection, but these are highly conserved in all mycobacterial species and are not specific for Mtb. An Mtb-specific enzyme molecule would be an ideal biomarker because enzyme-catalyzed signal amplification would help overcome the extremely slow growth rate of Mtb. Bertozzi et al. recently reported a sulfatase-activated probe for the in-gel assay of Mtb,[8] but this method is intended for use at reference laboratory level only.

We have been exploring BlaC, a hydrolase specifically expressed by Mtb, as a biomarker for Mtb detection.[9] BlaC is an Ambler class A β-lactamase that is highly conserved in Mtb clinical isolates, efficiently hydrolyzes β-lactam antibiotics, and is central to the biochemical mechanism responsible for pervasive β-lactam-antibiotic resistance.[10] Since the discovery of the first β-lactamase in 1940, a large number of β-lactamases have been identified that can hydrolyze a variety of β-lactam antibiotics, from penicillin to cephalosporins and carbapenems.[11] To assay the activity of β-lactamases, fluorogenic and luminogenic probes have been developed to take advantage of the high sensitivity of fluorescence and luminescence detection methods.[9a,^12^] However, these probes do not possess specificity for BlaC and can be hydrolyzed by many structurally homologous β-lactamases, such as TEM-1 Bla, the most common β-lactamase in Gram-negative bacteria.

The crystal structure of BlaC reveals a bigger and more flexible active site than most β-lactamases.[13] This important structural insight led us to hypothesize that BlaC could tolerate more bulky lactam substrates to provide specificity and thereby serve as a unique biomarker for Mtb detection. Previously, we introduced a methoxy substitution to the 7 position of the lactam ring and developed the BlaC-specific probe CDG-OMe (Figure 1 A).[9b] The catalytic efficiency of BlaC with CDG-OMe is 8900-fold higher than that of TEM-1 Bla. In this work, we further explored the effect on selectivity of substitutions at the 2 position of the β-lactam unit, with the central hypothesis that these substitutions would generate additional specificity for BlaC.

**Figure 1:**
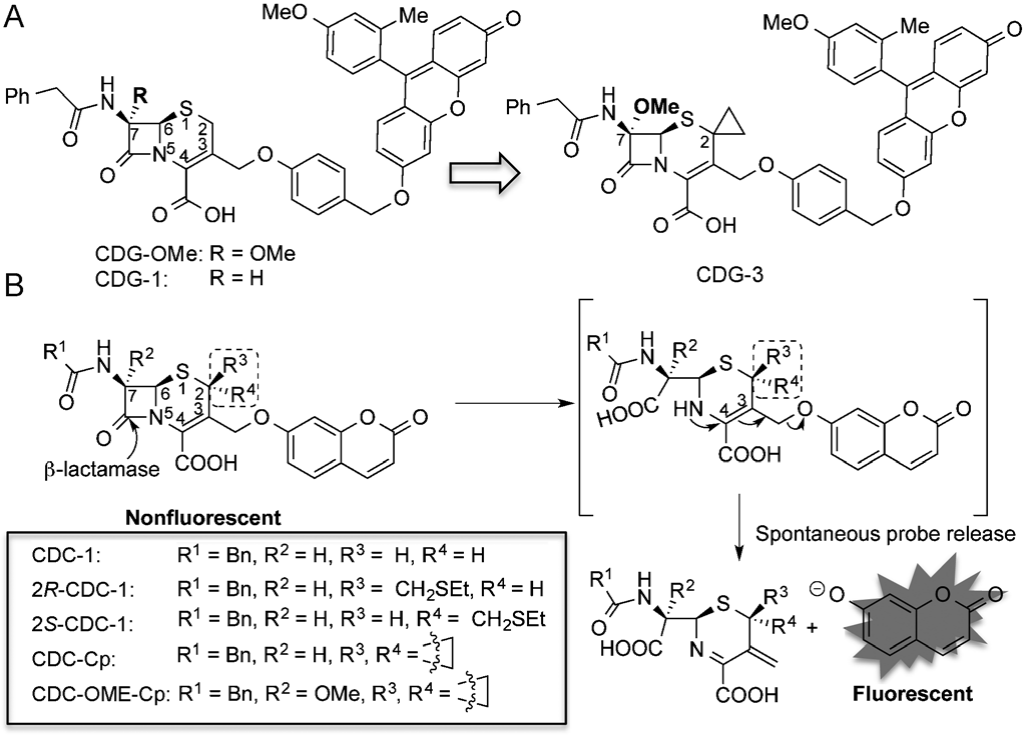
A) The structures of CDG-OMe, CDG-1, and CDG-3. B) Fluorescence detection of β-lactamase activity by using the CDC-series probes.

Fluorescence resonance energy transfer (FRET) has been used to develop ratiometric β-lactamase probes[12a] and may also be applied to the design of BlaC-specific probes. However, the need for dual excitation or emission presents additional demands for a corresponding POC device. In comparison, an “off/on” fluorogenic probe, with just a single excitation wavelength, is simpler. Therefore, we started with an umbelliferone-based, nonspecific β-lactamase fluorogenic substrate (CDC-1) and introduced a 2-ethylthiomethyl substitution that produced two epimers (*2R*-CDC-1, *2S*-CDC-1; Figure 1 B and Scheme S1 in the Supporting Information). Both CDC-1 analogues were hydrolyzed by BlaC and TEM-1 Bla, concurrently releasing the free umbelliferone (Figure 1 B) and generating a blue fluorescence signal.[14] The catalytic constant (*k*_cat_) and the Michaelis constant (*K*_m_) for BlaC and TEM-1 Bla are shown in Table S1 in the Supporting Information. In comparison to CDC-1, the substituent at the 2 position was well tolerated by BlaC (*k*_cat_/*K*_m_=1.1×10^5^ s^−1^ m^−1^ for *2R*-CDC-1 and 1.2×10^5^ s^−1^ m^−1^ for *2S*-CDC-1, versus 2.1×10^5^ s^−1^ m^−1^ for CDC-1) and the stereo conformation of the ethylthiomethyl group made little difference to the BlaC catalytic efficiency. However, the hydrolysis of *2S*-CDC-1 by TEM-1 Bla (*k*_cat_/*K*_m_=4×10^4^ s^−1^ m^−1^) was 10-fold less efficient than that of CDC-1 (*k*_cat_/*K*_m_=3.6×10^5^ s^−1^ m^−1^), while the catalytic efficiency was similar with *2R*-CDC-1 (*k*_cat_/*K*_m_=2.7×10^5^ s^−1^ m^−1^) and CDC-1. This result suggests that 2-substitution with the *S* conformation enhances the selectivity of the substrate for BlaC over TEM-1 Bla and that the *R* conformation does not have a major impact on the hydrolysis kinetics of BlaC and TEM-1 Bla.

We next examined the effect on the hydrolysis kinetics if both protons at the 2 position were substituted. Besides the potential selectivity for BlaC, there is an important advantage to replacing both of the protons, namely that it will avoid the well precedented, undesired isomerization of the 3,4-double bond to the 2,3-position, which would otherwise lead to the loss of probe activity (Figure 1 B and Figure S1 in the Supporting Information). A cylcopropyl substitution group was thus introduced to afford CDC-Cp (Figure 1 B and Scheme S2). As expected, CDC-Cp showed around 10-fold higher specificity for BlaC than TEM-1 Bla (*k*_cat_/*K*_m_=1.9×10^5^ s^−1^ m^−1^ for BlaC versus 2.2×10^4^ s^−1^ m^−1^ for TEM-1 Bla; see Table S1).

These results encouraged us to combine the 2-cyclopropyl and 7-OMe substitutions to give CDC-OMe-Cp (Figure 1 B and Scheme S2). CDC-OMe-Cp displayed remarkable specificity for BlaC: the catalytic efficiency of BlaC with this probe (*k*_cat_/*K*_m_=4.4×10^4^ s^−1^ m^−1^) is 6.3×10^4^ times higher than that of TEM-1 Bla (*k*_cat_/*K*_m_=0.7 s^−1^ m^−1^).

Fluorogenic probes that emit at a longer wavelength generally show improved detection sensitivity owing to reduced background signal. Therefore, a cyclopropyl substitution was introduced to the first generation BlaC-specific, Tokyo Green[15] based fluorogenic probe CDG-OMe to produce CDG-3 (Figure 1 A and Scheme S3).

Similar to CDG-OMe, CDG-3 generated a 214-fold increase in fluorescence after complete hydrolysis by BlaC (Figure S3). To our delight, CDG-3 showed higher selectivity for BlaC over TEM-1 Bla along with better sensitivity. As shown in Figure 2, CDG-3 generated a stronger fluorescent signal than CDG-OMe when incubated with the same amount of BlaC (10^−3^ pmol, Figure 2 A), thus demonstrating its enhanced sensitivity. Furthermore, the increased specificity of CDG-3 is demonstrated by its much slower hydrolysis by TEM-1 Bla compared to CDG-OMe (Figure 2 B); a 100 000-fold higher concentration of TEM-1 Bla still produced less fluorescent signal than BlaC. CDG-3 also showed little activity towards penicillinase (Pen) isolated from *Bacillus cereus* (Figure 2 C), while some activity was observed with CDG-OMe in the presence of a large amount of Pen (Figure 2 D, 10–100 pmol). Kinetic measurements confirmed the remarkable specificity of CDG-3 for BlaC (Table S1): the catalytic efficiency of BlaC (2.4×10^5^ s^−1^ m^−1^) for the hydrolysis of this probe was 120 000-fold higher than that of TEM-1 Bla (2 s^−1^ m^−1^) and 800 000-fold higher than of Pen (0.3 s^−1^ m^−1^). The stability of CDG-3 is high: the spontaneous hydrolysis rate (1.0×10^−7^ s^−1^) in MES buffer (0.1 m, pH 6.6) is lower than that of CDG-OMe (1.9×10^−7^ s^−1^).[9b]

**Figure 2:**
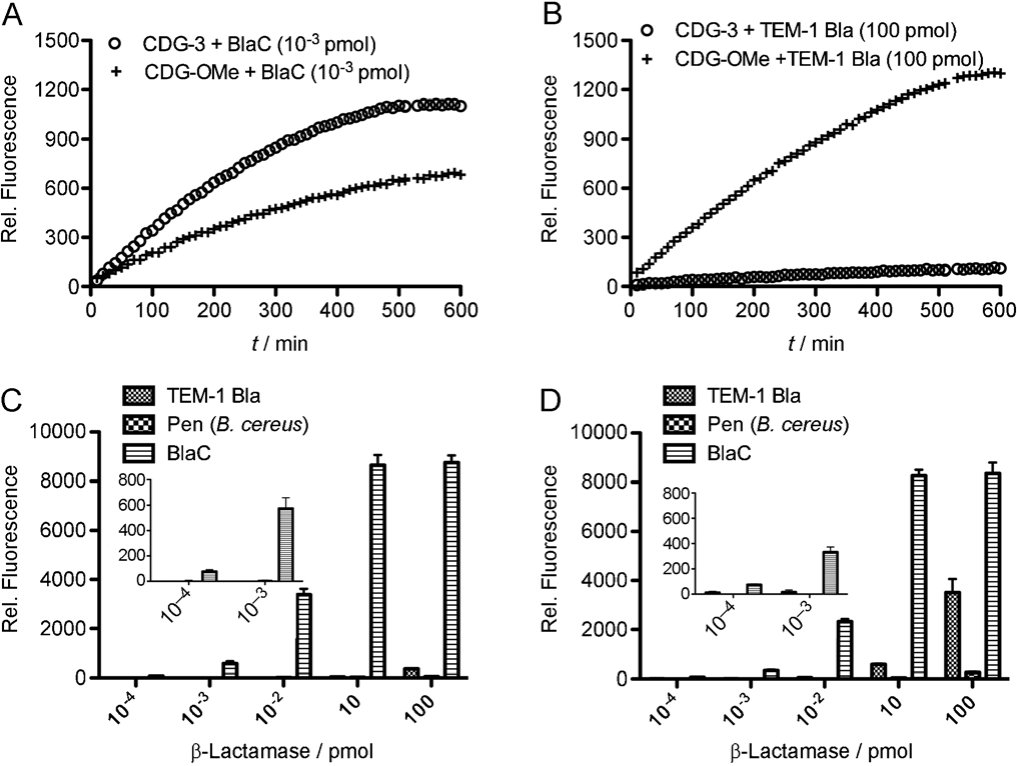
The β-lactamase selectivity of CDG-3 and CDG-OMe. A time course of fluorescence intensity produced by CDG-3 and CDG-OMe in the presence of BlaC (1 fmol; A) and TEM-1 Bla (100 pmol; B). Enhanced fluorescence intensity of CDG-3 (10 μm; C) and CDG-OMe (10 μm; D) after 3 h incubation with a series of diluted β-lactamases. Inserts show a magnified view of the intensity at low pmol quantities of β-lactamase. Data were collected in a 384-well plate with a total volume of 25 μL in each well. Fluorescence was measured with excitation at 490 nm and emission at 535 nm. Relative fluorescence represents the difference in fluorescence intensity with and without β-lactamase incubation. (C) and (D) show the average intensity of three replicate experiments. Error bars: standard deviation.

Next, we tested CDG-3 for the detection of *E. coli* transformed with *BlaC*[13] compared to a number of strains known for their high resistance to broad-spectrum cephalosporins: *K. pneumoniea* with extended-spectrum β-lactamase SHV-18*, E. cloacae* with AmpC β-lactamase, *K. pneumoniae* with the Class A carbapenemase KPC *(Klebsiella Pneumoniae* Carbapenemase*)*, and *E. coli* with the class B metallo-β-lactamase NDM-1.[16] *E. coli* transformed with *TEM-1 Bla* was used as a negative control. The fluorescence enhancements of the indicated probes (10 μm) were recorded after three hours of incubation across a dilution series of the β-lactamase-expressing bacteria and are shown in Figure 3 A. As expected, the nonspecific probe CDG-1 showed an “on” fluorescent signal with all of the bacteria and CDG-OMe showed much better selectivity for *E. coli* transformed with *BlaC* over other β-lactamase-expressing bacteria. However, at 10^5^ colony forming units (CFU), both AmpC- and KPC-expressing bacteria generated increased fluorescence emission with CDG-OMe: the fluorescence intensity ratio AmpC to BlaC (*I*_AmpC_/*I*_BlaC_) was 1:9 and *I*_KPC_/*I*_BlaC_=1/4. On the other hand, CDG-3 showed further enhanced specificity compared to CDG-OMe: in the presence of 10^5^ CFU, *I*_AmpC_/*I*_BlaC_ decreased to 1:130, and *I*_KPC_/*I*_BlaC_ dropped to 1:50. Figure 3 B shows fluorescence images of 10^6^ CFU of β-lactamase-expressing bacteria incubated with the CDG probes: CDG-3 gave a positive fluorescence signal only with BlaC and not with other β-lactamase-expressing bacteria.

**Figure 3:**
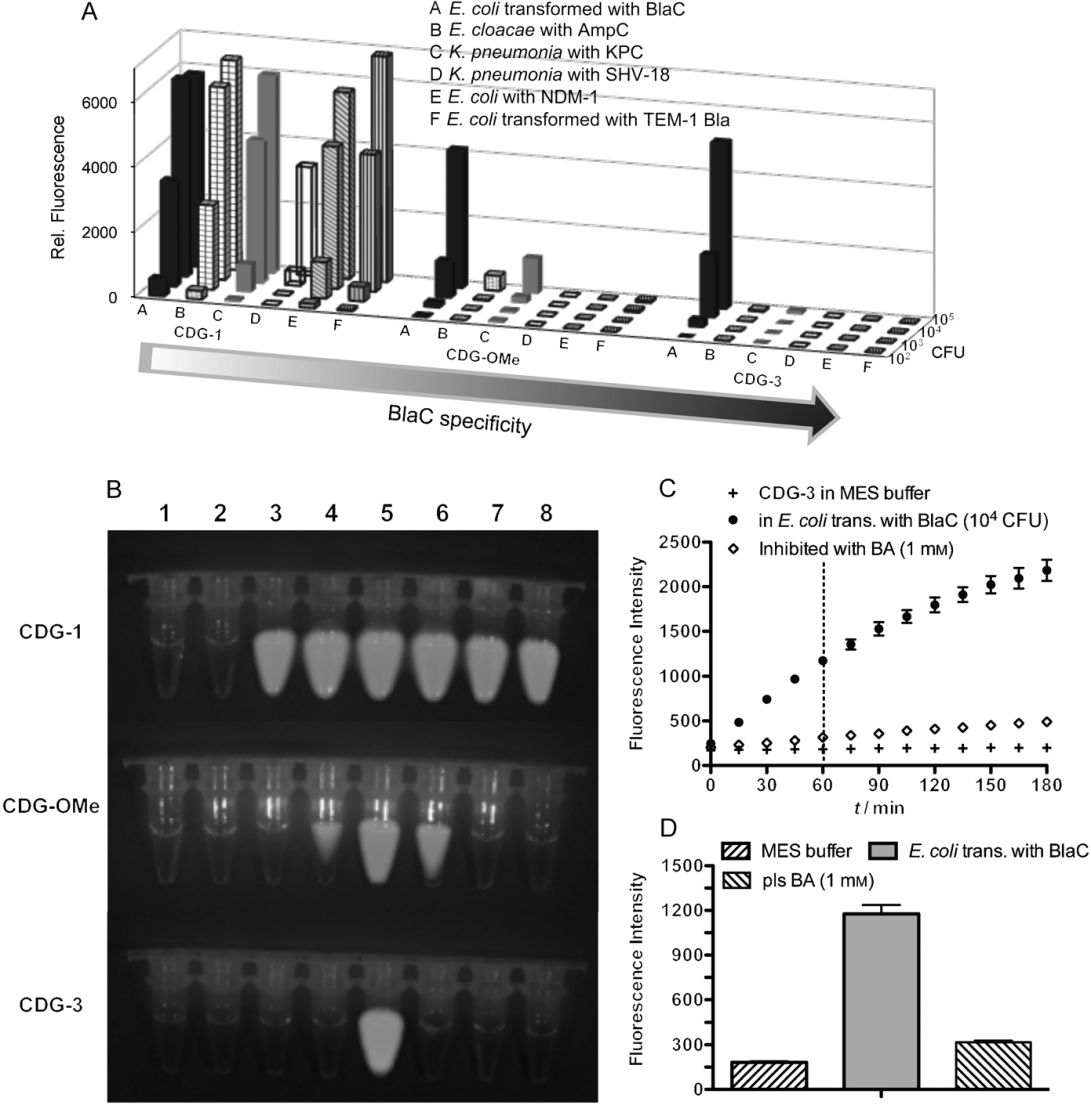
Evaluation of the CDG specificity of the probes for β-lactamases. A) Fluorescence intensity of CDG probes incubated with the indicated β-lactamase-expressing bacteria for 3 h at room temperature. B) Fluorescence imaging of β-lactamase-expressing bacteria (10^6^ CFU) after incubation with CDG probes (10 μm) at room temperature for 3 h (Ex: 500 nm; Em: 540 nm). From left to right: 1) Blank, 2) *E. coli*, 3) *K. pneumoniae* expressing SHV-18, 4) *E. cloacae* expressing AmpC, 5) *E. coli* transformed with *BlaC*, 6) *K. pneumoniae* expressing KPC, 7) *E. coli* expressing NDM-1, and 8) *E. coli* transformed with *TEM-1 Bla*. C) Time course of the fluorescence intensity of CDG-3 (10 μm) in the presence of MES buffer, and 10^4^ CFU *E. coli* transformed with *BlaC*. For the inhibition study, phenylboronic acid (BA, 1 mm) was added to the *BlaC*-transformed *E. coli* during incubation. D) Fluorescence intensity of CDG-3 after 1 h incubation with and without phenylboronic acid inhibition; Excitation at 490 nm and emission at 535 nm. Experiments were run in triplicate and the error bars show the standard deviation.

An inhibition study was performed to confirm that the fluorescent signal of CDG-3 was specific to the BlaC activity. As shown in Figure 3 C, D, CDG-3 showed little fluorescence in MES buffer, while fluorescence increased over time in the presence of *E. coli* expressing BlaC. By contrast, the CDG-3 fluorescence signal was significantly quenched after pre-incubating the bacteria with phenylboronic acid, a broad inhibitor that binds the serine in the active site for the hydrolysis of the β-lactam.[17]

The suitability of CDG-3 for detecting Mtb was first evaluated through testing with BCG, an attenuated *Mycobacterium bovis* strain, in unprocessed human sputum. Different amounts of BCG were added to Mtb-negative sputum containing high levels of other β-lactamase-expressing bacteria (Figure 4 A). Down to ten CFU (P<0.05), BCG can be detected readily in sputum (40 min, Figure 4 B), and the signal generated by BCG cleavage of CDG-3 in sputum is consistently higher than with sputum control over time (Figure 4 C). Moreover, the fluorescence emission generated by 10 CFU of BCG in sputum is significantly (P<0.05) higher than the fluorescence from the negative controls and even from 10^7^ CFU of other clinically prevalent bacteria that express β-lactamases, including *M. smegmatis, E. coli, P. aeruginosa* strain PA01, and methicillin-resistant *S. aureus* (MRSA) (Figure 4 D).

**Figure 4:**
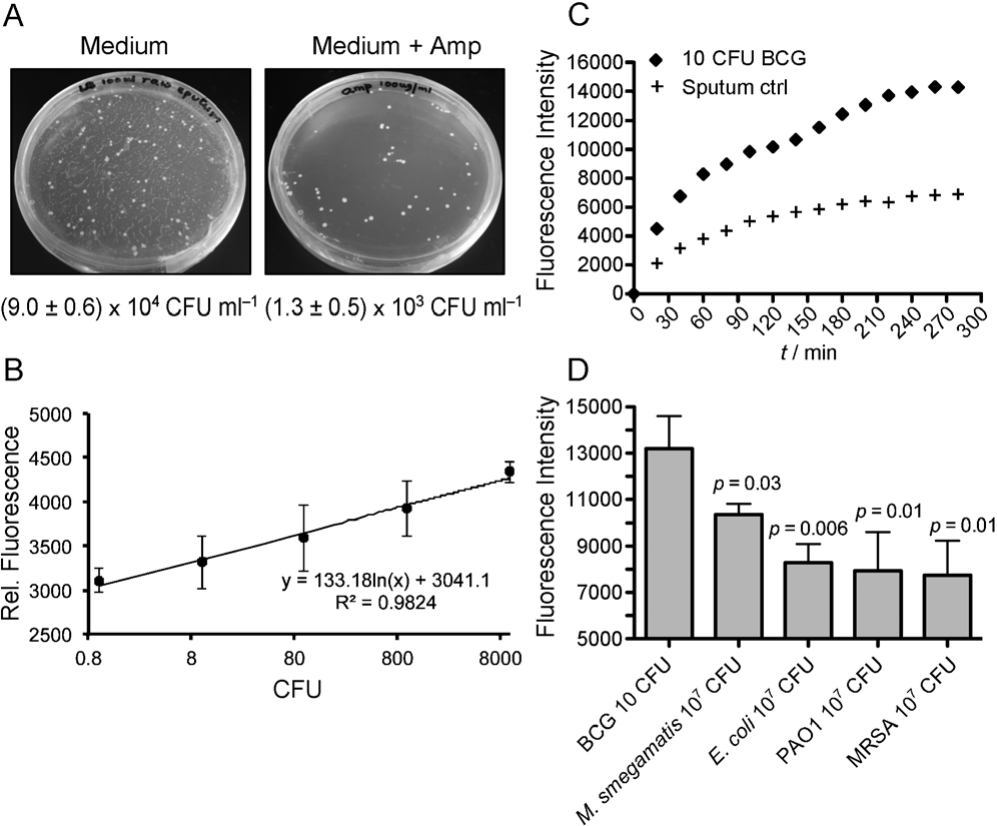
The detection of BCG added to unprocessed human sputum by using CDG-3. A) Bacterial abundance in Mtb-negative human sputum. Mtb-negative human sputa were plated on LB plates (left) to determine bacterial abundance and on LB plates supplemented with 100 μg mL^−1^ ampicillin (Amp; right) to determine the abundance of β-lactamase-producing bacteria. The samples were plated in duplicate and the average number and associated standard deviations are shown below each plate. B) The detection of BCG added to human sputum (40 mins). C) Time course of fluorescence intensity of CDG-3 with 10 CFU BCG added to human sputum and sputum control. D) The specificity of CDG-3 for detecting BCG (10 CFU) over indicated β-lactamase-expressing bacteria (10^7^ CFU) added to unprocessed human sputum. Data and error bars shown represent the means and standard deviations, respectively, of triplicate samples for all strains except BCG, for which there were six replicates. The *p*-values are for comparisons with BCG.

Finally, we applied CDG-3 to detect Mtb in clinical materials from suspected TB patients by using 50 blinded sputum samples. Sputum samples were mixed thoroughly with transport stabilization solution (TSS, 1:1) to achieve homogeneity. The homogenized sample was incubated for one hour at room temperature before fluorescence measurement. A TSS control comprising only TSS (no sputum) and a synthetic sputum control[18] were also included. Samples with fluorescence greater than twice that of the TSS control were considered positive in the CDG-3 assay. Both smear staining and culture tests were carried out for validation of the CDG-3 result. As summarized in Table 1, 100 % of the smear-positive and culture-positive samples (10/10) and 80 % of smear-negative and culture-positive samples (8/10) were detected as Mtb-positive by using CDG-3. Among 26 Mtb-negative (both smear-negative and culture-negative) samples, seven false positives were obtained, thus giving a specificity of 73 %. The reason for the false positives is presently unclear. Alternatively, these patients could be true Mtb positive but not identified by culture or smear assays. Follow-up of these patients to determine whether they subsequently become TB positive and further validation with a larger set of clinical samples would allow us to discriminate between these possibilities and improve diagnosis.

**Table 1:** CDG-3 test results with Mtb clinical specimens.[a]

CDG-3	Sm+ Cul+	Sm- Cul+	Sm- Cul-	Sensitivity[b]	Specificity[c]
(+)	10	8	7	90 %	73 %
(−)	0	2	19		
Total	10	10	26		

[a] Four smear-positive/culture-negative clinical samples were considered inconclusive before further validation and were thus excluded for this data analysis. Sm=smear test, cul=culture test.

[b] Sensitivity is calculated from the percentage of CDG-3 positive samples (10+8) in the total Mtb culture-positive samples (20);

[c] Specificity is calculated from the percentage of CDG-3-negative samples (19) in the Mtb-free samples [both smear and culture-negative (26)].

Smear microscopy is a century-old diagnostic test for pulmonary TB and is still the standard test for millions of suspected TB patients, even though it has a low sensitivity (20–80 %).[3c,d] About 17 % of transmission occurs from smear-negative TB patients, who present a great risk for the spread of TB to uninfected individuals.[19] Fluorescence microscopy can increase the sensitivity by about 10 %, but the higher equipment cost makes it less accessible in low-income countries, where more than 90 % of TB cases occur.[20] Mycobacterial culture is the reference standard and the most sensitive method for TB detection but it normally requires 2–8 weeks to obtain results, as well as specialized facilities and highly trained technicians owing to biosafety and contamination concerns. The Xpert technique enables rapid detection (2 h) with high sensitivity and specificity. In comparison, the sensitivity of the CDG-3 fluorescence assay matches it well: 100 % versus 99.7 % (Xpert) for both smear and culture-positive samples, and 80 % versus 76.1 % (Xpert) for smear-negative and culture-positive samples.[21]

While more clinical testing is required to further determine the specificity of the CDG-3 test, our results suggest that the CDG-3 assay can serve as a low-cost triage test in resource-limited areas, where TB prevalence is the highest and where high costs and instrument requirements are the major limitations to scaling up the current TB detection protocols. A recent analysis shows that a low-cost triage assay with 95 % sensitivity and 75 % specificity relative to Xpert can reduce the diagnostic cost by 34–43 % in India, South Africa, and Uganda and thus make screening all persons with presumptive TB more affordable.[22]

In summary, this work validates the use of the enzyme BlaC, which is specifically expressed by Mtb, as a biomarker for Mtb detection. The newly designed probe CDG-3 has a cyclopropane ring substitution at the 2 position in addition to the methoxy substitution at the 7 position and displays selectivity for BlaC over other β-lactamases, as confirmed by the detection of 10 CFU BCG from unprocessed human sputum in the presence of high levels of other β-lactamase-expressing clinically prevalent bacteria. In a pilot study with 50 TB patient samples, CDG-3 could be used to detect TB-positive samples with 90 % sensitivity and TB-negative samples with 73 % specificity. Further validation with a large set of clinical samples will help confirm CDG-3 as an important rapid, sensitive, and low-cost triage test for TB.
