# Supplementary material for: Fluorogenic Probes with Substitutions at the 2 and 7 Positions of Cephalosporin are Highly BlaC-Specific for Rapid Mycobacterium tuberculosis Detection
Source: Angew Chem Int Ed Engl. 2014 Jul 2;53(35):9360–4. doi: 10.1002/anie.201405243 (PMC4499257; doi:10.1002/anie.201405243)
Supplement: Supplementary file 1 [file anie0053-9360-sd1.pdf]

Angewandte Chemie
Eine Zeitschrift der Gesellschaft Deutscher Chemiker

Supporting Information
© Wiley-VCH 2014
69451 Weinheim, Germany

Fluorogenic Probes with Substitutions at the 2 and 7 Positions of Cephalosporin are Highly BlaC-Specific for Rapid *Mycobacterium tuberculosis* Detection**

*Yunfeng Cheng, Hexin Xie, Preeti Sule, Hany Hassounah, Edward A. Graviss, Ying Kong, Jeffrey D. Cirillo, and Jianghong Rao**

anie_201405243_sm_miscellaneous_information.pdf

Contents

1. General methods…………………………………………………………………....S01

2. Detection of mycobacteria in sputum ………………………………………………...S01

3. Figure S1………………………………………………………………………...….S02

4. Table S1 β-Lactamase kinetic parameters of fluorogenic probe ……...………………...S02

5. Figure S2 Maximum turn on of CDG-3 with BlaC ……...……………………………...S03

6. Figure S3 β-Lactamase selectivity of CDG-1…..……………………………………….S03

7. Figure S4, S5, S6 Enzymatic kinetics and stability tests …………………….…...........S04

8. Enzyme-inhibiting study …………………………………………………………........S06

9. Figure S7 Clinical sample testing with CDG-3……………………...…………………..S06

10. Probe synthesis and characterization……………………………………………......S07

References ……………………………………………………………………………S15

NMR spectra …………………………………………………………………………S17

1. General methods.

The purified TEM-1 β-lactamase was customarily prepared by the Biologics Process Development, Inc. (San Diego, California). Penicillinase from *Bacillus cereus* was purchased from Sigma-Aldrich (catalogue No. P0389). Recombinant *BlaC* was expressed in *E. coli* as previously described (1). All chemicals were purchased from commercial sources. *K. pneumonia with SHV-18, E. cloacae with AmpC, K. pneumoniae with KPC,* and *E. coli with NDM-1* lysates were kindly provided by Dr. Niaz Banaei from the Stanford Medical Center Microbiology Lab. Fluorescence spectra were obtained on a Fluoromax-3 spectrafluorometer (Jobin Yvon). Kinetic experiments were performed in a M1000 microplate reader (TECAN, research triangle park, NC). Analytical TLC was performed with 0.25 mm silica gel 60F plates with fluorescent indicator (254 nm). The ^{1}H and ^{13}C NMR spectra were taken on Varian 400 MHz magnetic resonance spectrometer. Data for ^{1}H NMR spectra are reported as follows: chemical shifts are reported as δ in units of parts per million (ppm) relative to chloroform-d (δ 7.26, s); multiplicities are reported as follows: s (singlet), d (doublet), t (triplet), q (quartet), dd (doublet of doublets), m (multiplet), or br (broadened); coupling constants are reported as a J value in Hertz (Hz); the number of protons (n) for a given resonance is indicated nH, and based on the spectral integration values. HPLC was performed on a Dionex HPLC System (Dionex Corporation) equipped with a GP50 gradient pump and an inline diode array UV-Vis detector. A reversed-phase C18 (Phenomenax, 5 μm, 10 x 250 mm or Dionex, 5 μm, 4.6 x 250 mm) column was used with a MeCN (B) / H_2O (A) gradient mobile phase containing 0.1% trifluoroacetic acid at a flow of 1 or 3 mL/min for the analysis.

2. Detection of mycobacteria in sputum.

Mtb var. *bovis* strain BCG was cultured in 7H9 medium with a 10% oleic acid albumin dextrose complex (OADC) and 0.25% Tween-80 until it reached the log phase (optical density at 600 nm (OD600) of 0.5–1). *E. coli, MRSA, P. aeruginosa strain PA01* and *M. smegmatis* were cultured in Luria–Bertani medium until OD600 = 0.5–1. After measuring the bacterial OD600, 10^7 c.f.u. of each bacterial strain was added into Eppendorf tubes. Bacteria were centrifuged, the supernatant removed and resuspended into the same medium (7H9 medium with 10% OADC) to normalize the autofluorescence from different media. A series of tenfold dilutions of BCG and *M. smegmatis* (10^7 c.f.u.), *E. coli* (10^7 c.f.u.), *Pseudomonas aeruginosa* (*PA01*, 10^7 c.f.u.), *Staphylococcus aureus* (*MRSA*, 10^7 c.f.u.) were added to pooled TB negative human sputum. Sputa were diluted 1:1 in in 200 mM MES buffer pH 6 plus 2% DTT, which constitutes transport stabilization solution (TSS), and incubated 0.3 generations (~40 min for BCG, 5 min for *M. smegmatis*, *E. coli*, *PA01* and *MRSA*) at room temperature. Reporter enzyme fluorescence solution (REFS; **CDG-3** in 200 mM MES**)**, was added and samples read immediately and after 40 min at room temperature in a 96-well plate using a Mithras LB940 plate reader at 490 nm (ex) and 535 nm (em).

For clinical samples testing using **CDG-3**, the obtained samples were mixed thoroughly with TSS (1:1) to achieve homogenization. The homogenized sample was incubated for one hour at room temperature before addition of REFS. The samples were read immediately using a Mithras LB940 plate reader at 490 (ex) and 535 (em), using a 100-point scan protocol. Each sample set was processed with two negative controls, a TSS control comprised of only TSS (no sputum) and the synthetic sputum control made as described previously (2).

3. CDC activity lost due to double bond isomerization.

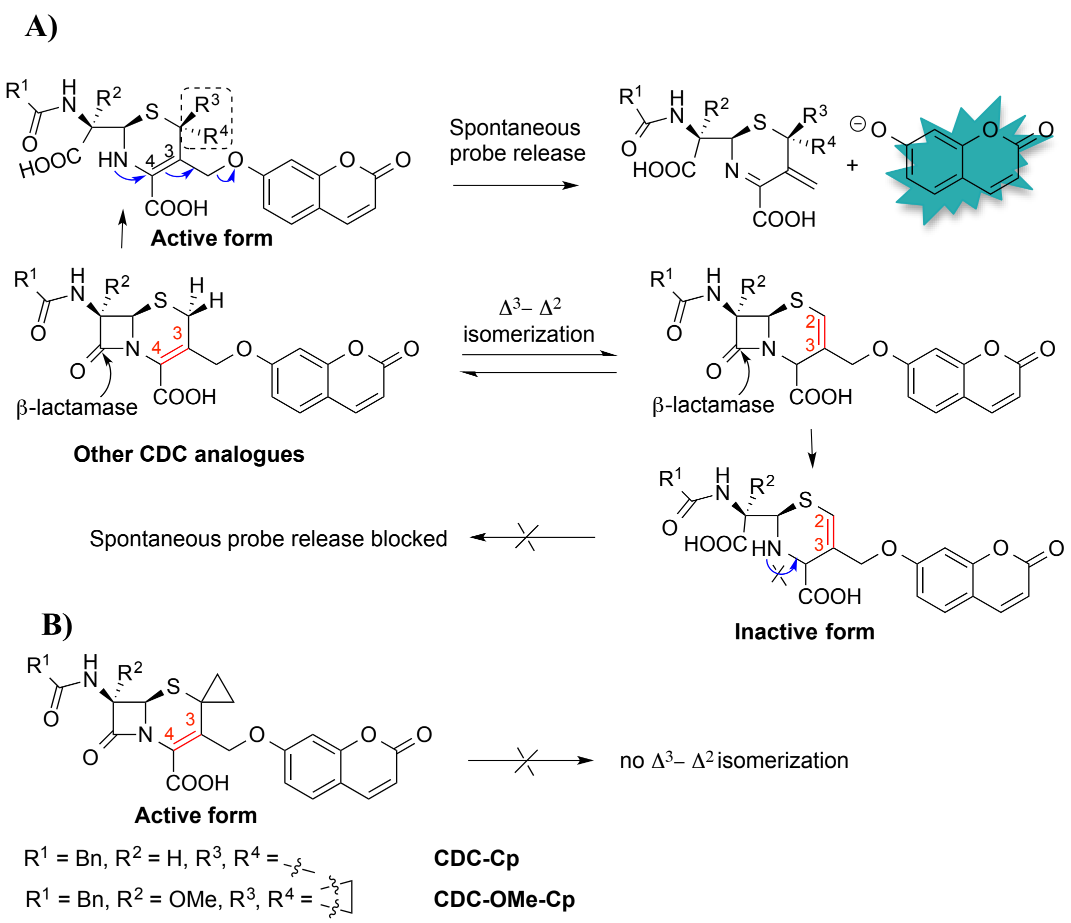

Figure S1. A) Schematic representation of inactive of CDC analogues due to undesirable double bond isomerization. B) Avoid of double bond isomerization of CDC-Cp and CDC-OMe-Cp with both 2-H substitutions

4. β-Lactamase kinetic parameters of fluorogenic probe.

Table S1. β-Lactamase kinetic parameters of fluorogenic probe

Name	BlaC			TEM-1 Bla			Penicillinase from *Bacillus cereus*			Spontaneous Hydrolysis Rate ($\times 10^{-7}$ s^{-1})
	K_m (μM)	k_{cat} (s^{-1})	k_{cat}/K_m ($s^{-1}M^{-1}$)	K_m (μM)	k_{cat} (s^{-1})	k_{cat}/K_m ($s^{-1}M^{-1}$)	K_m (μM)	k_{cat} (s^{-1})	k_{cat}/K_m ($s^{-1}M^{-1}$)	
**CDC-1**	63 ± 6[b]	13 ± 0.5[b]	2.1 x 10^5[b]	135 ± 16[b]	48 ± 3.8[b]	3.6 x 10^5[b]	ND	ND	ND	2.4[b]
**2*R*-CDC-1**	13.0 ± 1.0	1.5 ± 0.02	1.1 x 10^5	20.0 ± 0.04	5.5 ± 0.03	2.7 x 10^5	ND	ND	ND	5.1
**2*S*-CDC-1**	49.0 ± 2.9	5.9 ± 0.2	1.2 x 10^5	139.5 ± 12.3	5.6 ± 0.3	4.0 x 10^4	ND	ND	ND	2.9
**CDC-Cp**	13.9 ± 0.7	2.6 ± 0.03	1.9 x 10^5	69.5 ±7.9	1.5 ± 0.1	2.2 x 10^4	ND	ND	ND	4.5
**CDC-OMe-Cp**	217.9 ± 13.3	9.5 ± 0.4	4.4 x 10^4	75.2 ± 17.5	5.2 ± 0.5 x 10^{-5}	0.7	ND	ND	ND	2.5
**CDG-1**	2 ± 0.4[b]	1 ± 0.1[b]	5 x 10^5[b]	2 ± 0.2[b]	5 ± 0.1[b]	2.5 x 10^6[b]	7.8 ± 0.5	0.3 ± 0.01	3.9 x 10^5	6[b]
**CDG-OMe**	5 ± 0.3[b]	0.8 ±0.01[b]	1.6 x 10^5[b]	40 ± 4[b]	7 ± 0.6 x 10^{-4}[b]	18[b]	10.9 ± 4	4.6 ± 0.9 x 10^{-5}	4.3	1.9[b]
**CDG-3**	4.6 ± 0.5	1.1 ± 0.03	2.4 x 10^5	3.5 ± 0.4	7.0 ± 0.2 x 10^{-6}	2	12.2 ± 5.5	3.1 ± 0.6 x 10^{-6}	0.3	1.0

[a] Kinetic data were measured in MES buffer (100 mM, pH = 6.6, with 0.1% surfactant (3-[(3-cholamidopropyl) dimethylammonio]-1-propanesulfonate hydrate)) at room temperature (22°C). [b] ref 4. All data indicate averages of three replicate experiments. ND = not determined.

5. Maximum turn on of CDG-3 with BlaC.

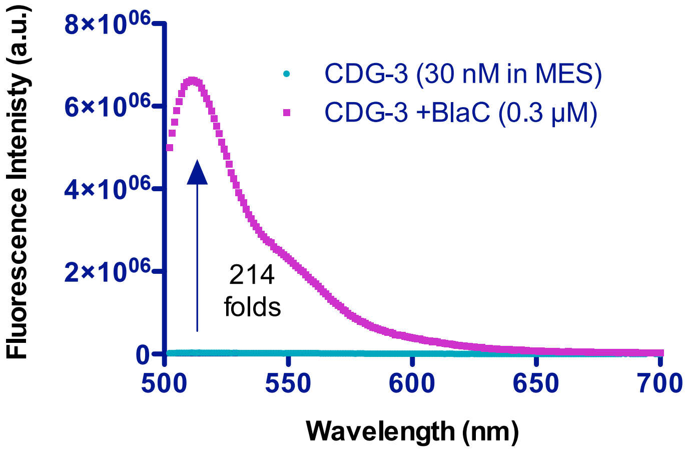

Figure S2. Fluorescent emission spectrum of CDG-3 (30 nM in MES buffer (0.1 M, pH = 6.6, with 0.1% surfactant (3-[(3-cholamidopropyl)dimethylammonio]-1-propanesulfonate hydrate)) before and after treatment with BlaC (0.3 μM) for 30 min (excitation: 490 nm).

6. β-Lactamase selectivity of CDG-1.

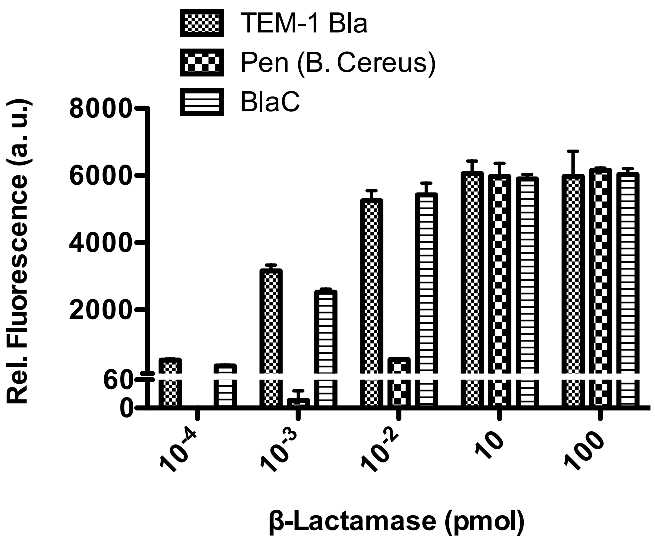

Figure S3. Enhanced fluorescence intensity of non-specific probe **CDG-1** (10 μM) by serial dilutions of ß-lactamases for 3 h. Data was collected in a 384-well plate with a total volume of 25 μL in each well. Fluorescence was measured with excitation at 490 nm and emission at 535 nm. Relative fluorescence represents the difference in fluorescence intensity with and without β-lactamase incubation. Error bars are ±SD.

7. Enzymatic kinetics and stability tests.

Enzymatic kinetics and stability tests of new analogues were determined as previously reported (3).

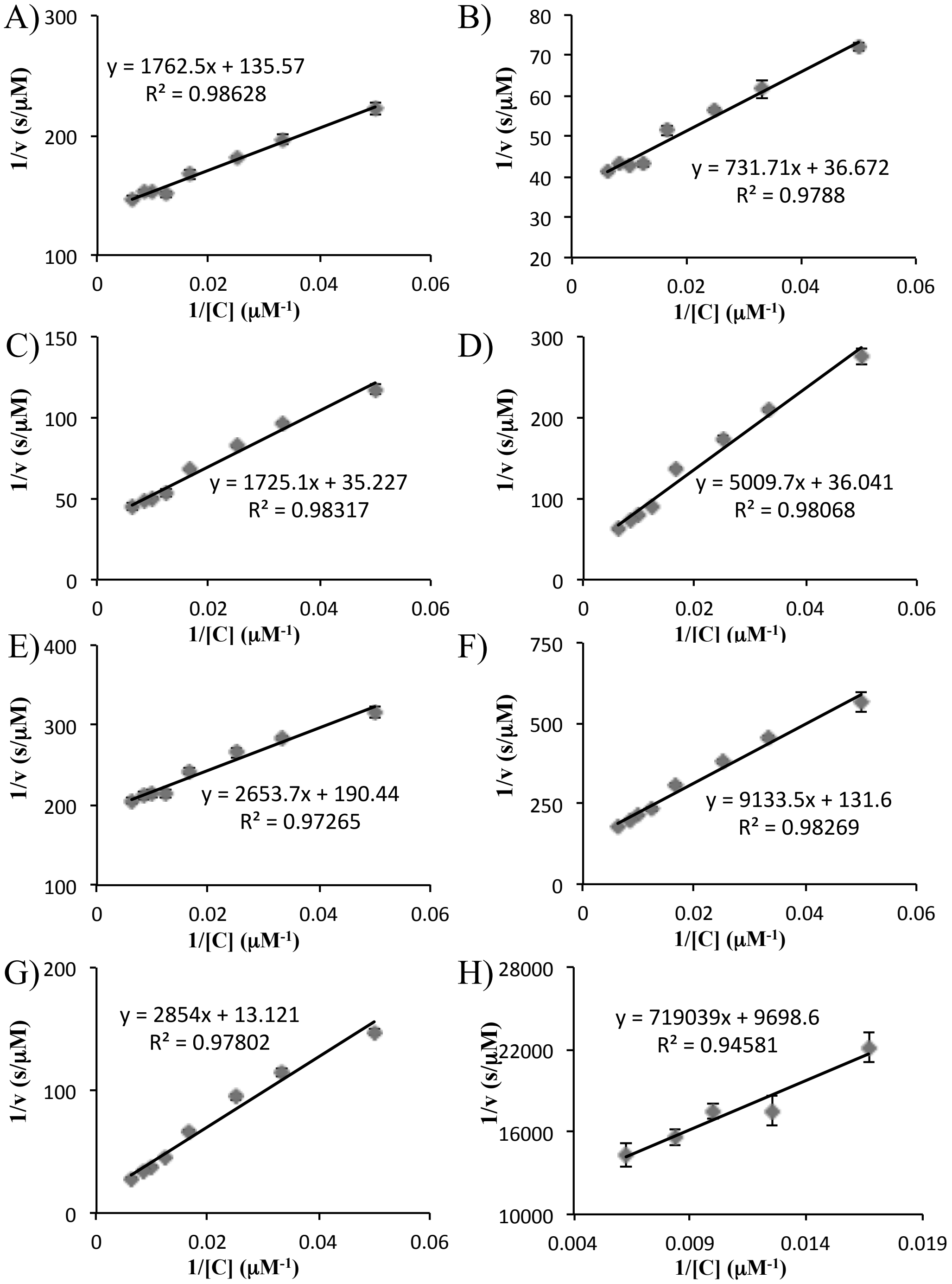

Figure S4. Lineweaver-Burke plot of β-lactamases with **CDC** series probes in MES (100 mM with 0.1% surfactant, pH 6.6). A) BlaC with **2*R*-CDC-1**; B) TEM-1 Bla with **2*R*-CDC-1**; C) BlaC with **2*S*-CDC-1**; D) TEM-1 Bla with **2*S*-CDC-1**; E) BlaC with **CDC-Cp**; F) TEM-1 Bla with **CDC-Cp**; G) BlaC with **CDC-OMe**, Cp; H) TEM-1 Bla with **CDC-OMe-Cp**. Error bars indicate the standard deviations of three replicate experiments.

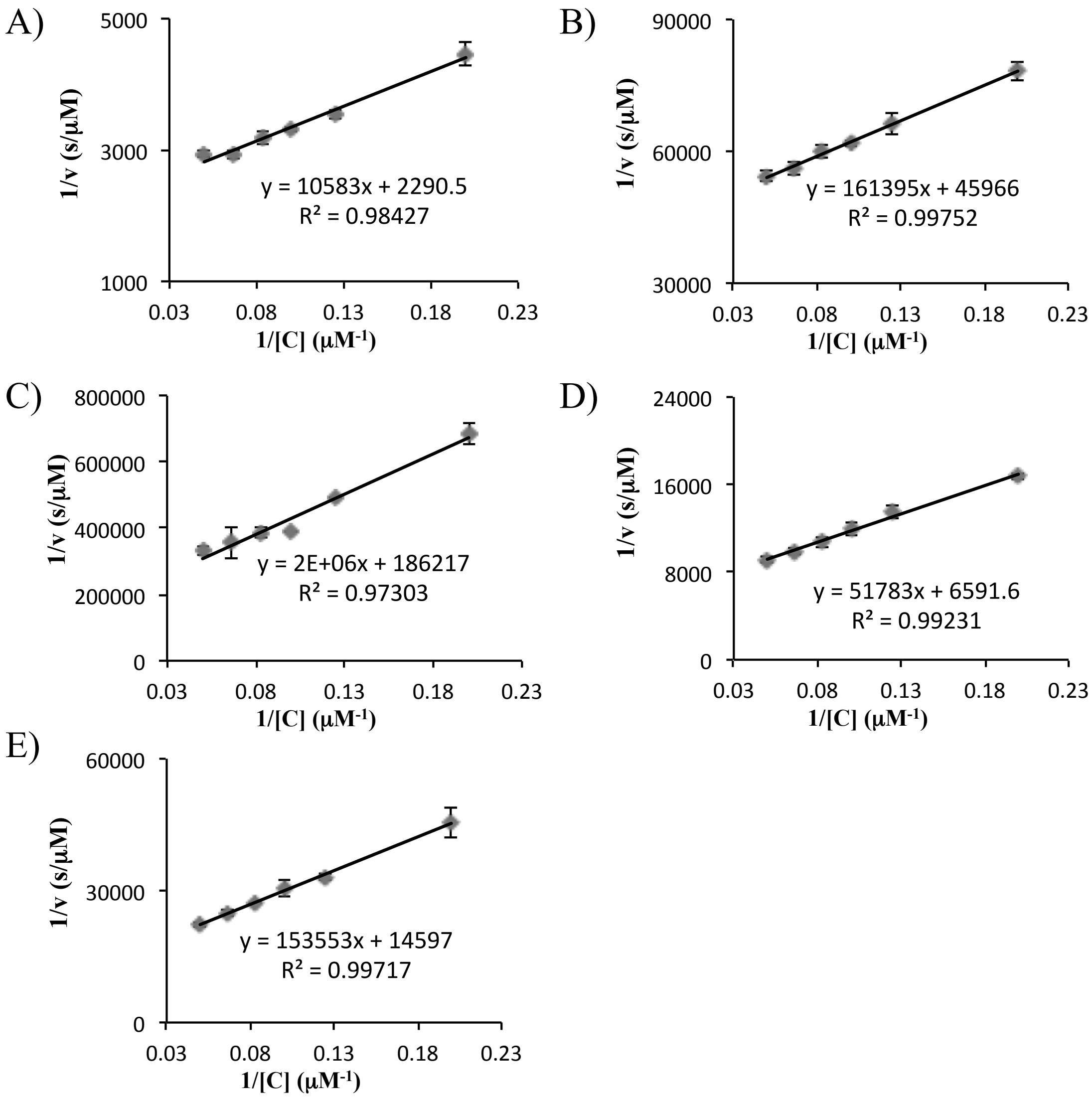

Figure S5. Lineweaver-Burke plot of β-lactamases with CDG series probes in MES (100 mM with 0.1% surfactant, pH 6.6). A) BlaC with **CDG-3**; B) TEM-1 Bla with **CDG-3**; C) Pen with **CDG-3**; D) Pen with **CDG-1**; E) Pen with **CDG-OMe**. Error bars indicate the standard deviations of three replicate experiments

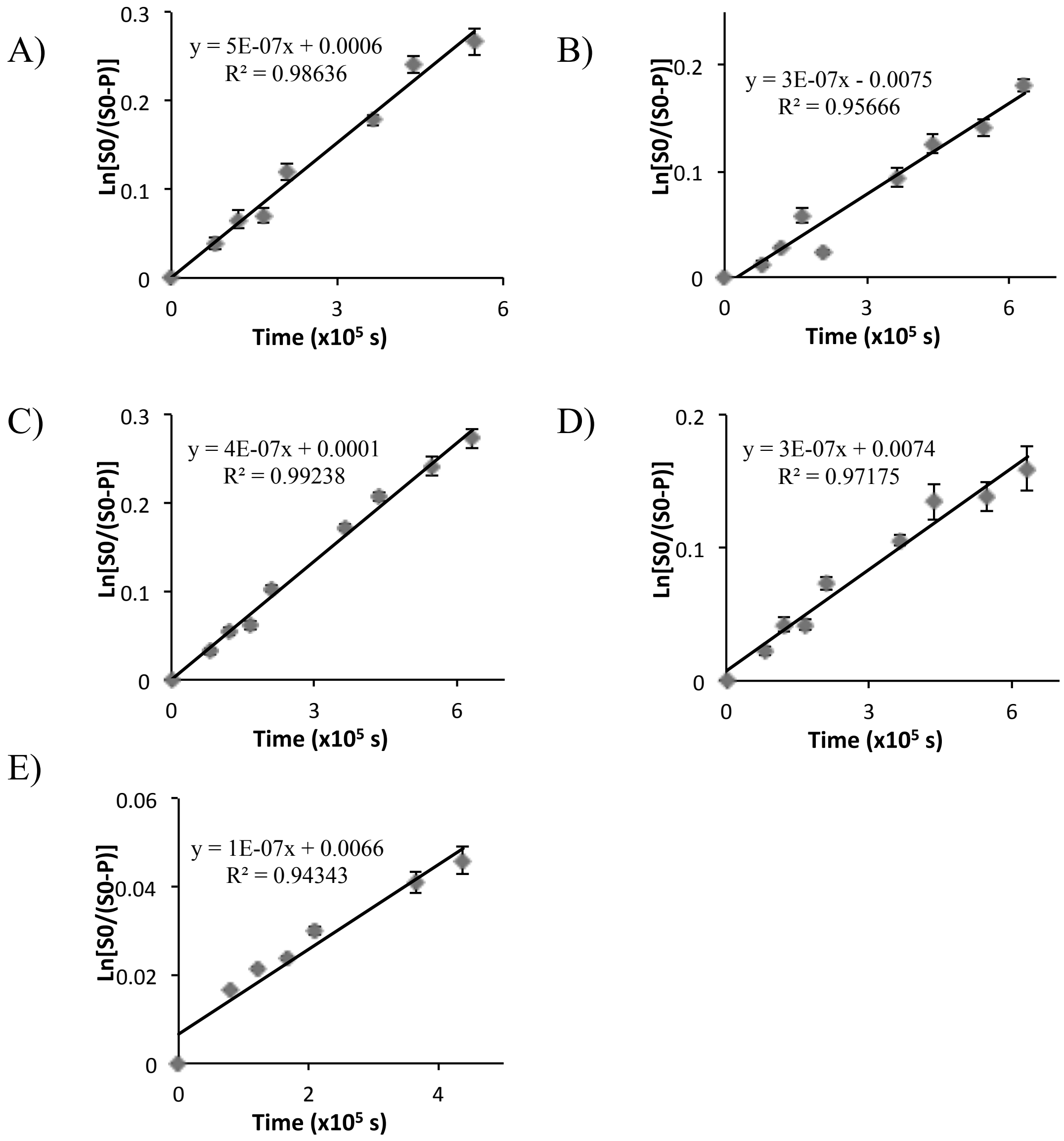

Figure S6. Stability measurement of fluorogenic probes in MES (100 mM with 0.1% surfactant, pH 6.6) at room temperature (22 ºC). A) ***2R*-CDC-1**; B) ***2S*-CDC-1**; C) **CDC-OMe**; D) **CDC-OMe-Cp**; E) **CDG-3**. Error bars indicate the standard deviations of three replicate experiments.

8. Enzyme-inhibiting study

To the well containing 10 µl MES buffer only, 10^4 c.f.u. *E. coli transformed with BlaC*, or pre-incubated of 10^4 c.f.u. *E. coli expressing BlaC* with phenylboronic acid (20 min, final concentration 1mM) was added 15 µl **CDG-3** (final concentration 10 µM). Fluorescence intensity was thus measured overtime in every 15 min. Excitation at 490 nm and emission at 535 nm.

9. Clinical sample testing with CDG-3

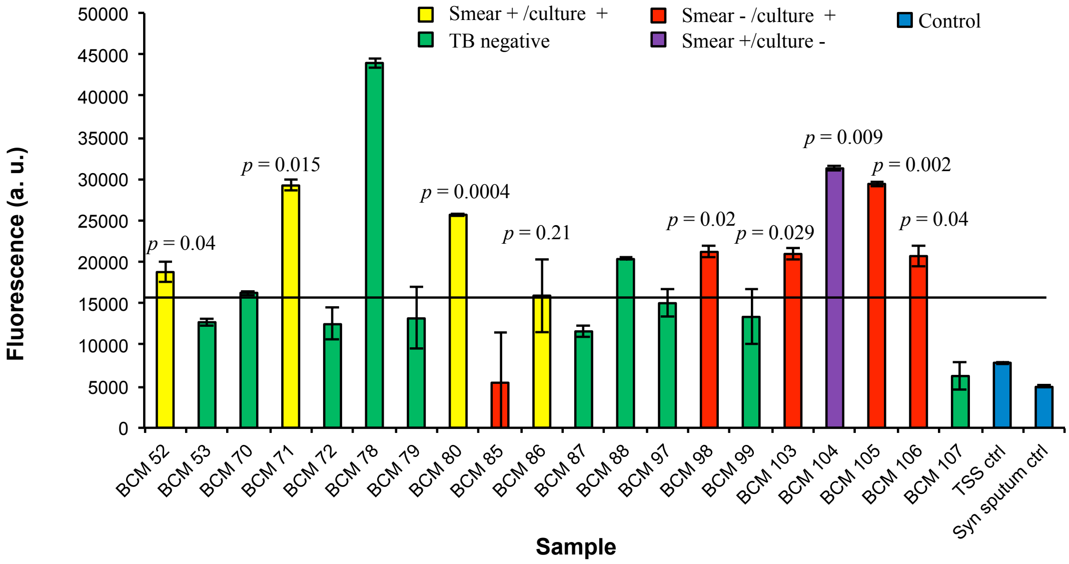

Figure S7. Representative twenty of total 50 clinical samples in a blinded fashion using **CDG-3** in a reporter enzyme fluorescence (REF) assay after 1h incubation at room temperature. Samples with fluorescence greater than twice that of the buffer (transport stabilization solution, TSS) control were considered positive (threshold shown by black horizontal line). Data and error bars shown represent the mean and standard deviation of top two values from a 100 point well scan for each sample. *p*-values are comparison versus the TSS control.

10. Probe synthesis and characterization.

10.1. Preparation of ***2R*-CDC-1 and *2S*-CDC-1.**

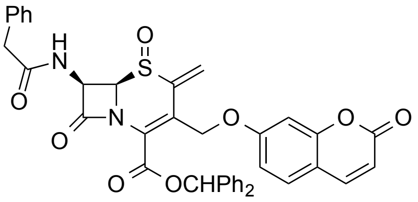

11

Benzhydryl(7*R*,6*R*)-7-(2-phenylacetamido)-3-(((2-oxo-2H-chromen-7-yl)oxy)methyl)-2-methylene-1-oxide-3-cephem-4-carboxylate (11). Compound **11** was prepared according to a method modified from literature(3). To a solution of **10** (802 mg, 1.19 mmol)(4) and formaldehyde (37% aqueous solution, 1 mL, 12.3 mmol) in DMF (7.5 mL) and dioxane (7.5 mL) was added dimethylamine hydrochloride (194 mg, 2.4 mmol) and the reaction mixture was heated to 55 ºC for 6 h. After starting material disappeared (monitored by TLC), solvent and excess reagent were removed by Rota-Vap. Purification by flash chromatography on silica gel column afforded the titled compound **11** (661mg, 84%). ^{1}H NMR (400 MHz, d^6-DMSO) δ 8.67 (d, *J* = 8.3 Hz, 1H), 7.99 (d, *J* = 9.6 Hz, 1H), 7.57 (d, *J* = 8.7 Hz, 1H), 7.42 – 7.11 (m, 15H), 6.98 (s, 1H), 6.88 (d, *J* = 2.3 Hz, 1H), 6.80 (dd, *J* = 8.6, 2.4 Hz, 1H), 6.41 (s, 1H), 6.31 (d, *J* = 9.5 Hz, 1H), 6.24 (s, 1H), 6.00 (dd, *J* = 8.3, 5.0 Hz, 1H), 5.15 (d, *J* = 5.0 Hz,

1H), 5.11 (d, J = 11.5 Hz, 1H), 4.87 (d, J = 11.5 Hz, 1H), 3.69 (d, J = 14.0 Hz, 1H), 3.57 (d, J = 14.0 Hz, 1H); ^{13}C NMR (101 MHz, d^6-DMSO) δ 171.80, 164.51, 162.98, 161.54, 160.94, 160.64, 155.84, 144.98, 142.42, 140.11, 139.75, 136.44, 130.59, 130.04, 129.83, 129.15, 128.99, 128.56, 127.70, 127.60, 127.26, 127.05, 116.85, 113.46, 113.42, 102.06, 79.90, 69.74, 64.27, 59.47, 42.05, 36.46, 31.44; HRMS: Calculated for $C_{39}H_{30}N_2NaO_8S^+$ ([M+Na]$^+$): 709.1615; Found: 709.1597.

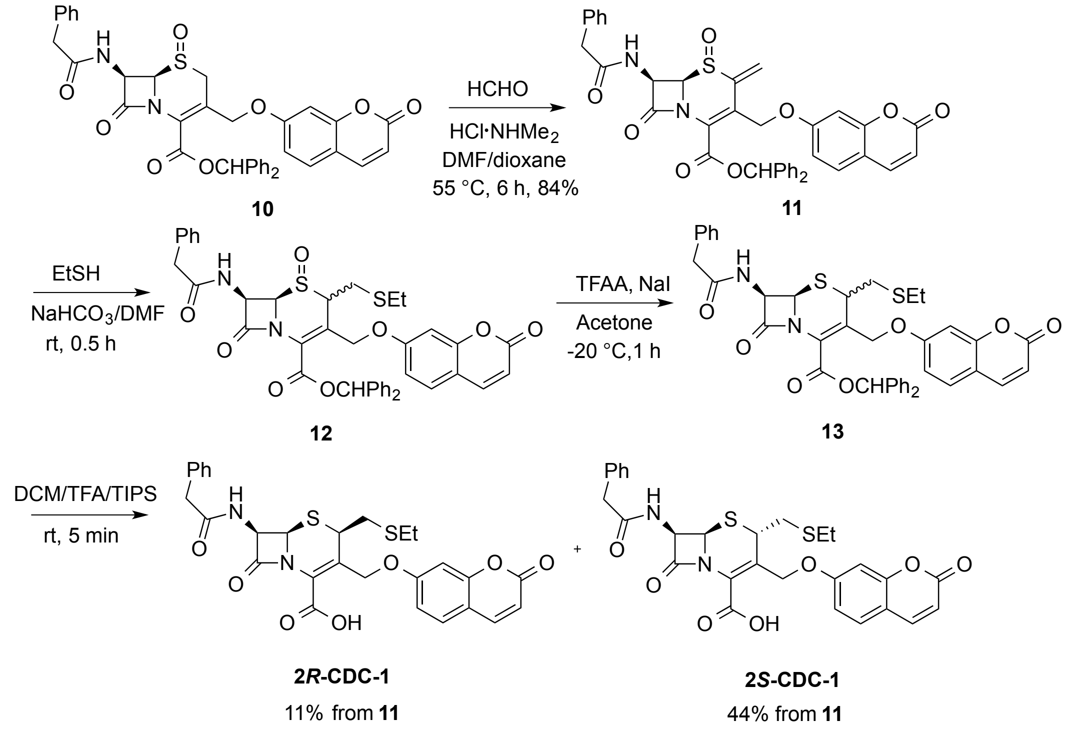

Scheme S1. Synthesis of **2*R*-CDC-1** and **2*S*-CDC-1**

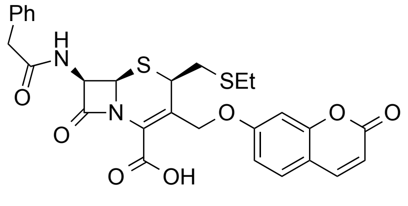

2*R*-CDC-1

(7*R*, 6*R*, 2*R*)-7-(2-phenylacetamido)-3-(((2-oxo-2H-chromen-7-yl)oxy)methyl)-2-((ethylthio)methyl)- 3-cephem-4-carboxylic acid (*2R*-CDC-1). To a mixture of **11** (68.9 mg, 0.1 mmol) and $NaHCO_3$ (4 mg, 0.05 mmol) in DMF (0.5 mL) was added ethanethiol (10 μL, 0.14 mmol) and the reaction was stirred at room temperature for 0.5 h. After starting material **11** disappeared, compound **12** as crude product was obtained after flash chromatography on a short silica gel column. Under Ar, trifluoroacetic anhydride (71 μL, 0.5 mmol) was added slowly to a mixture of sodium iodide (150 mg, 1 mmol) and compound **12** in acetone (anhydrous, 7 mL) at -40 ºC, the reaction mixture was then stirred at -20 ºC for 1 h. After the disappearance of **12**, reaction was cooled down to -78 ºC and ethyl acetate was added, followed by sodium bicarbonate saturated aqueous solution. The reaction temperature was then allowed to room temperature. Organic layer was separated and the aqueous layer was extracted with ethyl acetate (20 mL x 2). The combined organic layers were dried over $MgSO_4$. Crude compound **13** was then obtained after solvent was removed (the

diastereomeric ratio was determined by ^{1}H NMR spectrum of the crude product to be 77.8: 22.2), which was then subjected to a mixture CH_2Cl_2/TFA/TIPS (1/0.9/0.05 mL) at room temperature for 5 minutes. Pure **2*R*-CDC-1** (6 mg, 11% for 3 steps) and **2*S*-CDC-1** (25 mg, 44% for 3 steps) were obtained after HPLC purification on a C18 column. **2*R*-CDC-1**: ^{1}H NMR (400 MHz, d^6-DMSO) δ 9.15 (d, *J* = 8.0 Hz, 1H), 7.99 (d, *J* = 9.5 Hz, 1H), 7.64 (d, *J* = 8.6 Hz, 1H), 7.35 – 7.15 (m, 5H), 6.98 (d, *J* = 2.3 Hz, 1H), 6.94 (dd, *J* = 8.6, 2.4 Hz, 1H), 6.30 (d, *J* = 9.5 Hz, 1H), 5.62 (dd, *J* = 8.0, 4.7 Hz, 1H), 5.23 (d, *J* = 4.8 Hz, 1H), 5.09 (d, *J* = 12.5 Hz, 1H), 4.94 (d, *J* = 12.5 Hz, 1H), 4.30 (dd, *J* = 9.1, 4.4 Hz, 1H), 3.56 (d, *J* = 14.0 Hz, 1H), 3.51 (d, *J* = 14.1 Hz, 1H), 3.26 (dd, *J* = 13.7, 4.5 Hz, 1H), 2.79 (dd, *J* = 13.6, 9.2 Hz, 1H), 2.54 (q, *J* = 7.4 Hz, 2H), 1.13 (t, *J* = 7.4 Hz, 3H); HRMS: Calculated for $C_{28}H_{26}N_2NaO_7S^+$ ([M+Na]$^+$): 589.1074; Found: 589.1057.

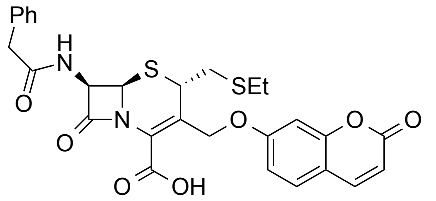

2*S*-CDC-1

(7*R*,6*R*,2*S*)-7-(2-phenylacetamido)-3-(((2-oxo-2H-chromen-7-yl)oxy)methyl)-2-((ethylthio)methyl)-3-cephem-4-carboxylic acid (2*S*-CDC-1). ^{1}H NMR (400 MHz, d^6-DMSO) δ 9.19 (d, *J* = 8.4 Hz, 1H), 7.99 (d, *J* = 9.5 Hz, 1H), 7.64 (d, *J* = 8.7 Hz, 1H), 7.35 – 7.16 (m, 5H), 7.06 (d, *J* = 2.3 Hz, 1H), 6.98 (dd, *J* = 8.6, 2.4 Hz, 1H), 6.30 (d, *J* = 9.5 Hz, 1H), 5.76 (dd, *J* = 8.3, 4.9 Hz, 1H), 5.26 (d, *J* = 4.9 Hz, 1H), 5.00 (s, 2H), 3.86 (dd, *J* = 10.1, 3.1 Hz, 1H), 3.54 (d, *J* = 13.9 Hz, 1H), 3.48 (d, *J* = 13.9 Hz, 1H), 3.13 (dd, *J* = 14.1, 3.1 Hz, 1H), 2.83 (dd, *J* = 14.1, 10.1 Hz, 1H), 2.53 (q, *J* = 7.4 Hz, 2H), 1.10 (t, *J* = 7.4 Hz, 3H); ^{13}C NMR (101 MHz, d^6-DMSO) δ 171.62, 165.30, 163.71, 161.79, 160.91, 155.95, 144.97, 136.38, 130.31, 129.71, 128.94, 128.28, 127.22, 122.78, 113.44, 102.08, 66.74, 60.19, 54.51, 42.24, 41.36, 36.92, 26.29, 15.43; HRMS: Calculated for $C_{28}H_{26}N_2NaO_7S^+$ ([M+Na]$^+$): 589.1074; Found: 589.1062.

10.2. Preparation of **CDC-Cp and CDC-OMe-Cp.**

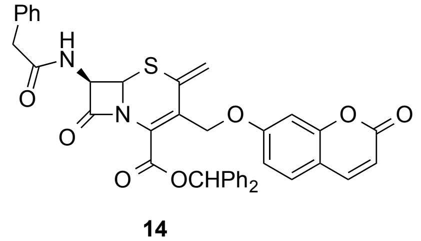

14

Benzhydryl(7*R*,6*R*)-7-(2-phenylacetamido)-3-(((2-oxo-2H-chromen-7-yl)oxy)methyl)-2-methylene-3-cephem-4-carboxylate (14) Under Ar, trifluoroacetic anhydride (350 μL, 2.48 mmol) was added slowly to a mixture of sodium iodide (735 mg, 14.7 mmol) and compound**11** in acetone (anhydrous, 20 mL) at -40 ºC, the reaction mixture was then stirred at -20 ºC for 1 h. The reaction was cooled down to -78 ºC and ethyl acetate was added, followed by sodium bicarbonate saturated aqueous solution. The reaction temperature was then raised to room temperature. Organic layer was separated and the aqueous layer was

extracted with ethyl acetate (20 mL x 2). The combined organic layers were dried over $MgSO_4$. Compound **14** (314 mg, 95%) was then obtained by flash chromatography purification. ^1H NMR (400 MHz, $CDCl_3$) δ 7.63 (d, *J* = 9.5 Hz, 1H), 7.43 – 7.15 (m, 17H), 6.98 (s, 1H), 6.70 (dd, *J* = 8.6, 2.4 Hz, 1H), 6.61 (d, *J* = 2.3 Hz, 1H), 6.28 (d, *J* = 9.5 Hz, 1H), 5.90 (dd, *J* = 8.8, 4.8 Hz, 1H), 5.81 (s, 1H), 5.68 (s, 1H), 5.11 (d, *J* = 4.8 Hz, 1H), 4.98 (d, *J* = 10.8 Hz, 1H), 4.77 (d, *J* = 10.8 Hz, 1H), 3.68 (d, *J* = 16.1 Hz, 1H), 3.62 (d, *J* = 16.1 Hz, 1H); ^{13}C NMR (101 MHz, $CDCl_3$) δ 171.47, 163.71, 161.27, 161.23, 155.86, 143.53, 138.87, 138.66, 133.70, 130.69, 129.72, 129.52, 129.06, 128.75, 128.72, 128.65, 128.37, 128.09, 128.06, 127.10, 126.91, 123.38, 122.29, 113.79, 113.27, 113.01, 101.85, 80.21, 63.25, 60.54, 56.99, 43.49; HRMS: Calculated for $C_{39}H_{30}N_2NaO_7S^+$ ($[M+Na]^+$): 693.1666; Found: 693.1658.

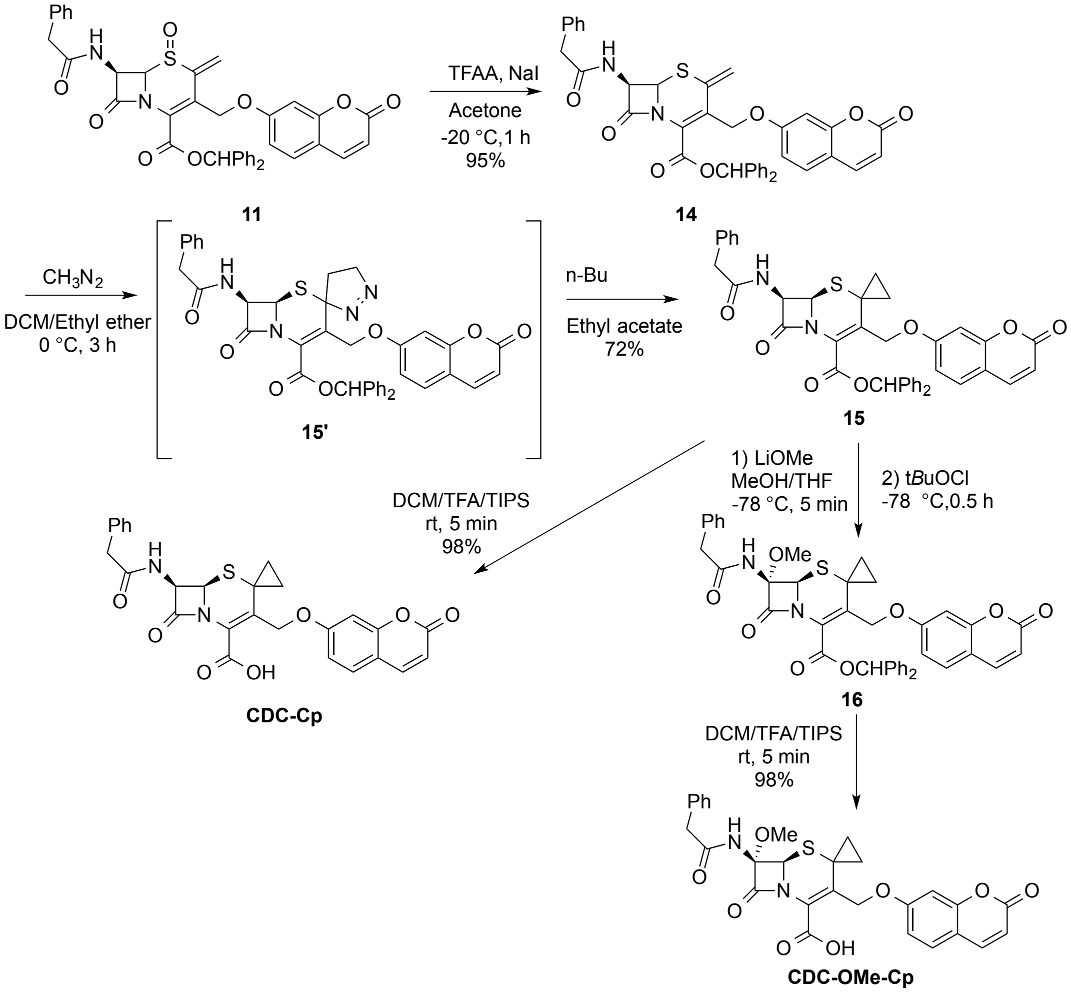

Scheme S2. Synthesis of **CDC-Cp** and **CDC-OMe-Cp.**

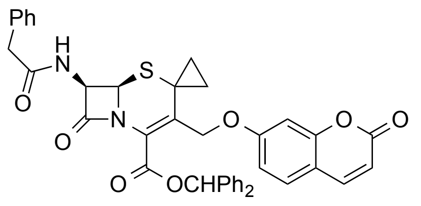

15

Benzhydryl(7*R*,6*R*)-7-(2-phenylacetamido)-3-(((2-oxo-2H-chromen-7-yl)oxy)methyl)-2-cyclopropyl-3-cephem-4-carboxylate (15)(5). **Caution!** Diazomethane is carcinogenic and potentially explosive! Erlenmeyer flasks (no ground joints) were used in reaction. To a

solution of **14** (314 mg, 0.47 mmol) in CH_2Cl_2 (20 mL) at 0 ºC was added diazomethane(6) (4.7 mmol) in ethyl ether (20 mL) and the reaction was kept at 0 ºC for 3 h. After the reaction completed, acetic acid was added slowly at 0 ºC to quench the excess diazomethane (until the reaction solution turned from yellow to colorless). The resulting mixture was washed with water and $NaHCO_3$ aqueous solution subsequently, and dried over $MgSO_4$. After filtration and Rota-Vap, the residue was dissolved in ethyl acetate and heated to 50 ºC for 30 min until all intermediate **15′** transformed (monitored by TLC). Purification by flash chromatography on silica gel column afforded the titled compound **15 (**232 mg, 72%). ^{1}H NMR (400 MHz, $CDCl_3$) δ 7.62 (d, *J* = 9.5 Hz, 1H), 7.42 – 7.14 (m, 17H), 6.96 (s, 1H), 6.66 (dd, *J* = 8.6, 2.4 Hz, 1H), 6.52 (d, *J* = 2.3 Hz, 1H), 6.28 (d, *J* = 9.5 Hz, 1H), 6.10 (d, *J* = 8.7 Hz, 1H), 5.92 (dd, *J* = 8.8, 4.9 Hz, 1H), 5.22 (d, *J* = 4.8 Hz, 1H), 4.37 (d, *J* = 10.8 Hz, 1H), 4.29 (d, *J* = 10.8 Hz, 1H), 3.66 (d, *J* = 16.1 Hz, 1H), 3.60 (d, *J* = 16.2 Hz, 1H), 1.52 – 1.40 (m, 2H), 1.34 (dd, *J* = 9.3, 4.6 Hz, 1H), 1.03 – 0.95 (m, 1H); ^{13}C NMR (101 MHz, $CDCl_3$) δ 165.06, 161.37, 161.19, 160.97, 155.83, 143.46, 139.08, 138.82, 133.70, 129.71, 129.52, 129.05, 128.69, 128.67, 128.57, 128.26, 128.10, 128.08, 127.10, 127.04, 113.85, 113.28, 112.82, 101.76, 79.82, 62.91, 60.09, 59.70, 43.53, 22.28, 21.29, 13.78; HRMS: Calculated for $C_{40}H_{32}N_2NaO_7S^+$ ($[M+Na]^+$): 707.1822; Found: 707.1808.

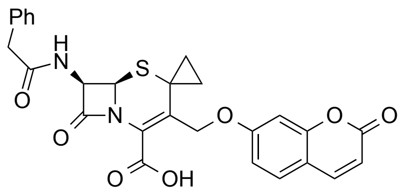

CDC-Cp

(7*R*,6*R*)-7-(2-phenylacetamido)-3-(((2-oxo-2H-chromen-7-yl)oxy)methyl)-2-cyclopropyl-3-cephem-4-carboxylic acid (CDC-Cp). Compound **15** (92 mg, 0.134 mmol) was subjected to a mixture of CH_2Cl_2/TFA/TIPS (1/0.9/0.05 mL) at room temperature for 5 minutes. After removing the solvent, the residue was then purified by flash chromatography on silica gel column to afford the titled compound **CDC-Cp** 68 mg, 98%). ^{1}H NMR (400 MHz, d^6-DMSO) δ 9.16 (d, *J* = 8.4 Hz, 1H), 7.98 (d, *J* = 9.5 Hz, 1H), 7.62 (d, *J* = 8.0 Hz, 1H), 7.40 – 7.16 (m, 5H), 7.00 (s, 1H), 6.94 (d, *J* = 8.6 Hz, 1H), 6.29 (d, *J* = 9.6 Hz, 1H), 5.79 (dd, *J* = 7.5, 4.8 Hz, 1H), 5.28 (d, *J* = 4.8 Hz, 1H), 4.60 (d, *J* = 11.0 Hz, 1H), 4.41 (d, *J* = 11.0 Hz, 1H), 3.53 (d, *J* = 14.0 Hz, 1H), 3.47 (d, *J* = 14.0 Hz, 1H), 1.52-1.40 (m, 2H), 1.39-1.29 (m, 1H), 0.99-0.90 (m, 1H); ^{13}C NMR (101 MHz, d^6-DMSO) δ 171.60, 165.64, 163.77, 161.70, 160.92, 155.97, 144.97, 136.39, 130.19, 129.99, 129.69, 128.93, 127.20, 124.98, 113.43, 113.33, 101.98, 63.67, 60.44, 59.69, 42.24, 21.81, 21.03, 13.41; HRMS: Calculated for $C_{27}H_{22}N_2NaO_7S^+$ ($[M+Na]^+$): 541.1040; Found: 541.1033.

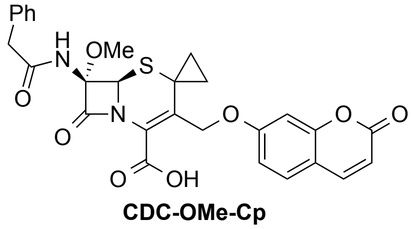

CDC-OMe-Cp

(7S, 6R)-7-(2-phenylacetamido)-7-methoxy-3-(((2-oxo-2H-chromen-7-yl)oxy)methyl)-

2-cyclopropyl-3-cephem-4-carboxylic acid (CDC-OMe-Cp)(4, 7). Under Ar, a solution of lithium methoxide (19.4 mg, 0.5 mmol) in methanol (anhydrous, 0.6 mL) was added dropwise to a solution of **15** (137 mg, 0.2 mmol) in anhydrous THF (3 mL) at -78 ºC and the reaction was stirred for 5 min. *Tert*-butyl hypochlorite (36 μL, 0.32 mmol) was then added dropwise and the mixture was stirred at the same temperature for half an hour. Anhydrous THF (5 mL) was added to dilute and the resulting solution was poured in one port to an aqueous solution containing ammonium chloride and sodium bisulfite, extracted with ethyl acetate (15 mL x 3) and dried over $MgSO_4$. Compound **16** was obtained as crude product after solvent was removed, which was then treated with a mixture CH_2Cl_2/TFA/TIPS (1/0.9/0.05 mL) at room temperature for 5 minutes. The titled compound **CDC-OMe-Cp** (45 mg, 39% for 2 steps) was obtained after HPLC purification. ^{1}H NMR (400 MHz, d^6-DMSO) δ 9.56 (s, 1H), 7.97 (d, J = 9.5 Hz, 1H), 7.61 (d, J = 8.7 Hz, 1H), 7.37 – 7.15 (m, 5H), 7.01 (d, J = 2.4 Hz, 1H), 6.93 (dd, J = 8.6, 2.4 Hz, 1H), 6.28 (d, J = 9.5 Hz, 1H), 5.31 (s, 1H), 4.54 (d, J = 11.5 Hz, 1H), 4.39 (d, J = 11.4 Hz, 1H), 3.59 (d, J = 14.2 Hz, 2H), 3.54 (d, J = 14.2 Hz, 2H), 3.35 (s, 3H), 1.52 – 1.42 (m, 1H), 1.41 – 1.31 (m, 1H), 1.29 – 1.18 (m, 1H), 1.00 – 0.87 (m, 1H); ^{13}C NMR (101 MHz, d^6-DMSO) δ 172.42, 163.49, 161.65, 161.32, 160.92, 155.95, 144.96, 136.16, 130.19, 129.84, 129.61, 128.92, 127.22, 125.32, 113.42, 113.36, 101.97, 96.08, 64.58, 63.69, 53.17, 42.31, 21.86, 21.03, 18.54, 13.02; HRMS: Calculated for $C_{28}H_{24}N_2NaO_8S^+$ ($[M+Na]^+$): 571.1146; Found: 571.1125.

10.3. Preparation of **CDG-3.**

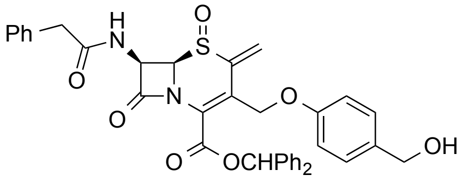

4

Benzhydryl(7*R*,6*R*)-7-(2-phenylacetamido)-3-((4-(hydroxymethyl)phenoxy)methyl)-2-methylene-1-oxide-3-cephem-4-carboxylate (4). To a solution of **3**(4) (500 mg, 0.785 mmol) and formaldehyde (37% aqueous solution, 0.64 mL, 7.9 mmol) in DMF (6 mL) and dioxane (6 mL) was added dimethylamine hydrochloride (128 mg, 1.57 mmol) and the reaction mixture was heated to 55 ºC for 6 h. Solvents and excess reagent were removed by Rota-Vap. Purification by flash chromatography on silica gel column afforded the titled compound **4** (418 mg, 82%). ^{1}H NMR (400 MHz, d^6-DMSO) δ 8.64 (d, J = 8.2 Hz, 1H), 7.46 – 7.21 (m, 14H), 7.17 (d, J = 8.3 Hz, 2H), 6.99 (s, 1H), 6.76 (d, J = 8.6 Hz, 2H), 6.36 (s, 1H), 6.22 (s, 1H), 5.98 (dd, J = 8.3, 5.0 Hz, 1H), 5.13 (d, J = 4.9 Hz, 1H), 5.07 (s, 1H), 5.02 (d, J = 11.2 Hz, 1H), 4.74 (d, J = 11.5 Hz, 1H), 4.40 (s, 2H), 3.68 (d, J = 14.1 Hz, 1H), 3.56 (d, J = 14.0 Hz, 1H); ^{13}C NMR (101 MHz, d^6-DMSO) δ 171.79, 164.51, 160.71, 157.36, 142.63, 140.13, 139.88, 136.44, 135.99, 130.42, 129.82, 129.19, 129.12, 128.99, 128.70, 128.61, 128.50, 127.71, 127.25, 127.23, 127.13, 117.54, 114.99, 79.88, 69.68, 63.84, 63.14, 59.36, 42.04. HRMS: Calculated for $C_{37}H_{32}N_2NaO_7S^+$ ($[M+Na]^+$): 671.1822; Found: 671.1818.

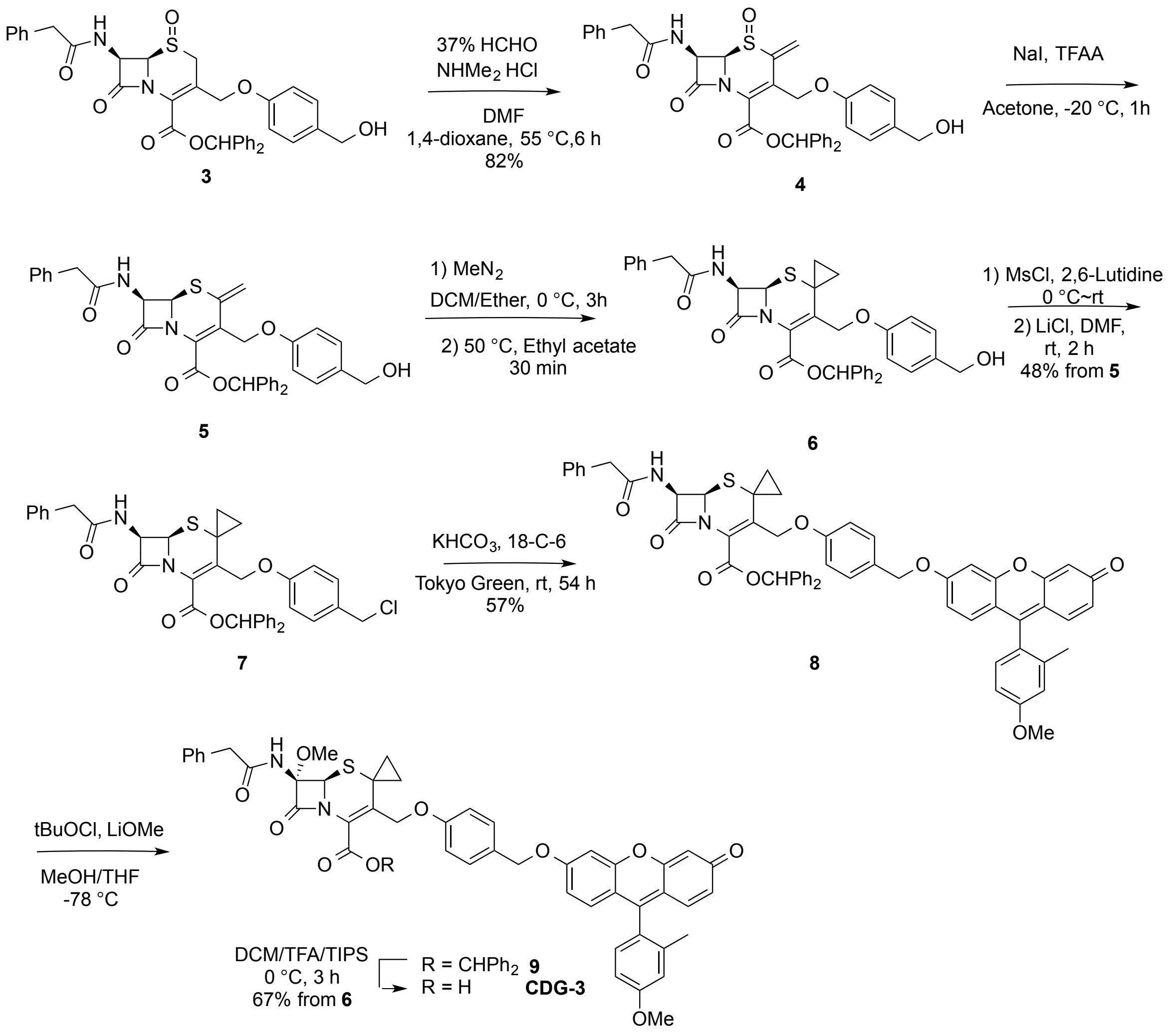

Scheme S3. Synthesis of **CDG-3**

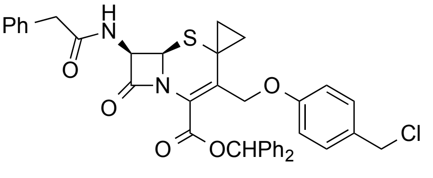

7

Benzhydryl(7*R*,6*R*)-7-(2-phenylacetamido)-3-((4-(chloromethyl)phenoxy)methyl)-2-cyclopropyl-3-cephem-4-carboxylate (7). Under Ar, trifluoroacetic anhydride (304 μL, 2.2 mmol) was added slowly to a mixture of sodium iodide (645 mg, 4.3 mmol) and **2** (275 mg, 0.43 mmol) in acetone (anhydrous, 10 mL) at -40 ºC, the reaction mixture was then stirred at -20 ºC for 1 h. Reaction mixture was cooled down to -78 ºC and ethyl acetate was added, followed by sodium bicarbonate saturated aqueous solution. The reaction temperature was then allowed to rise to room temperature. Organic layer was separated and the aqueous layer was extracted with ethyl acetate (20 mL x 2). The combined organic layers were dried over $MgSO_4$ and the solvent was removed to afford **5.** To a solution of **5** in CH_2Cl_2 (4 mL) at 0 ºC was added fresh prepared diazomethane[5] (**Caution!** Diazomethane is carcinogenic and potentially explosive! 4.9 mmol) in ethyl ether (10 mL) and the reaction was kept at 0 ºC for 4 h. After the reaction completed, acetic acid (0.5 mL) was added slowly at 0 ºC to quench the excess diazomethane (until the reaction solution turned from yellow to colorless). The resulting mixture was washed with water and $NaHCO_3$ aqueous solution subsequently, and

dried over $MgSO_4$. After filtration and Rota-Vap, the residue was dissolved in ethyl acetate (15 mL) and heated to 50 ºC for 40 min. After solvent removal, the residue was purified by flash chromatography on silica gel column by using hexane/ethyl acetate (5/1-3/1) to afford the crude compound **6**. To a solution of **6** obtained above, 2,6-lutidine (93 μL, 0.8 mmol) in DMF (anhydrous, 1.5 mL) at 0 ºC, was added slowly methanesulfonyl chloride (42 μL, 0.54 mmol). The reaction was stirred at 0 ºC for 0.5 h and room temperature for 1 h. Lithium chloride (230 mg, 5.4 mmol) was then added and stirred at room temperature for another 2 h. Ethyl acetate and water were added. After separation, the aqueous layer was further extracted with ethyl acetate twice. The combined organic layer was washed with water and brine, and dried over $MgSO_4$. After solvents removal, the residue was then purified by flash chromatography on silica gel column by using hexane/ethyl acetate (3/1) as eluting solvents to afford the titled compound **7** (136 mg, 48% for 3 steps from **4**). ^{1}H NMR (400 MHz, $CDCl_3$) δ 7.45 – 7.11 (m, 17H), 6.99 (s, 1H), 6.65 (d, J = 8.7 Hz, 2H), 6.24 (d, J = 8.8 Hz, 1H), 5.91 (dd, J = 8.8, 4.8 Hz, 1H), 5.21 (d, J = 4.8 Hz, 1H), 4.55 (s, 2H), 4.37 (d, J = 11.0 Hz, 1H), 4.32 (d, J = 11.0 Hz, 1H), 3.64 (d, J = 16.0 Hz, 1H), 3.59 (d, J = 16.0 Hz, 1H), 1.60 – 1.43 (m, 2H), 1.36 – 1.25 (m, 1H), 1.00 – 0.89 (m, 1H); ^{13}C NMR (101 MHz, $CDCl_3$) δ 171.48, 165.15, 161.60, 158.05, 139.25, 138.92, 133.85, 130.71, 130.36, 129.71, 129.46, 128.71, 128.65, 128.53, 128.25, 128.10, 128.00, 127.71, 127.20, 114.88, 79.75, 62.81, 60.07, 59.79, 46.37, 43.49, 22.50, 21.50, 13.80; HRMS: Calculated for $C_{38}H_{33}ClN_2NaO_5S^+$ ($[M+Na]^+$): 687.1691; Found: 687.1714.

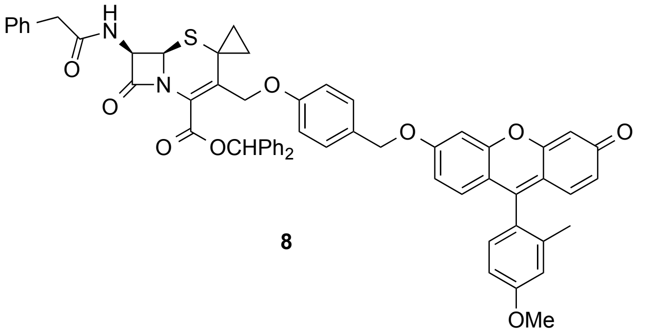

8

Benzhydryl(7*R*,6*R*)-7-(2-phenylacetamido)-3-((4-(((9-(4-methoxy-2-methylphenyl)-3-oxo-3H-xanthen-6-yl)oxy)methyl)phenoxy)methyl)-2-cyclopropyl-3-cephem-4-carboxylate (8). A mixture of Tokyo Green(8) (56 mg, 0.17 mmol), potassium bicarbonate (34 mg, 0.34 mmol) and 18-crown-6 (30 mg, 0.11 mol) in DMF (anhydrous, 0.2 mL) were stirred at rt for 5 min, then **5** (73.6 mg, 0.11 mol) was added and the resulting mixture was stirred in dark at rt for 54 h. Purification by flash chromatography on silica gel column provided pure **8** (61.5 mg, 57%). ^{1}H NMR (400 MHz, $CDCl_3$) δ 7.41 – 7.14 (m, 17H), 7.12 – 7.01 (m, 4H), 6.96 (s, 1H), 6.94 – 6.83 (m, 3H), 6.71 (d, J = 8.7 Hz, 2H), 6.67 (dd, J = 9.7, 1.9 Hz, 1H), 6.60 (s, 1H), 6.26 (d, J = 8.8 Hz, 1H), 5.91 (dd, J = 8.8, 4.9 Hz, 1H), 5.21 (d, J = 4.8 Hz, 1H), 5.10 (s, 2H), 4.38 (d, J = 11.1 Hz, 1H), 4.34 (d, J = 11.0 Hz, 1H), 3.89 (s, 3H), 3.65 (d, J = 16.1 Hz, 1H), 3.60 (d, J = 16.0 Hz, 1H), 2.03 (s, 3H), 1.57 – 1.44 (m, 2H), 1.34 – 1.27 (m, 1H), 1.12 – 0.99 (m, 1H); ^{13}C NMR (101 MHz, $CDCl_3$) δ 185.11, 171.45, 165.11, 164.04, 161.54, 160.73, 159.36, 158.26, 155.17, 139.26, 138.97, 138.05, 133.83, 131.22, 130.61, 130.07, 129.70, 129.64, 129.45, 128.67, 128.66, 128.44, 128.33, 128.17, 128.09, 127.98, 127.83, 127.18, 124.59, 118.83, 116.31, 115.31, 115.00, 114.83, 111.82, 105.72, 101.44, 79.72, 70.79, 62.80, 60.06, 59.79, 55.63, 43.48, 22.47, 21.51, 20.27, 13.82; HRMS: Calculated for $C_{59}H_{49}N_2O_9S^+$ ($[M+H]^+$): 961.3153; Found: 961.3158.

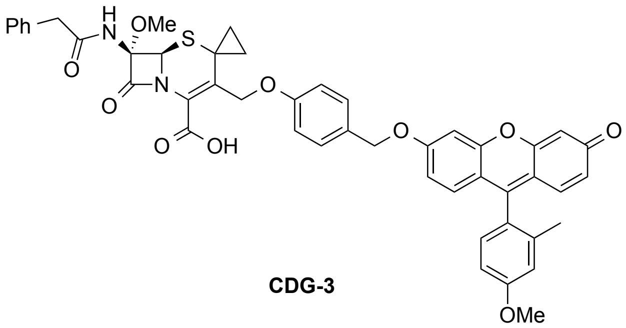

CDG-3

(7*S*,6*R*)-7-(2-phenylacetamido)-7-methoxy-3-((4-(((9-(4-methoxy-2-methylphenyl)-3-oxo-3H-xanthen-6-yl)oxy)methyl)phenoxy)methyl)-2-cyclopropyl-3-cephem-4-carboxylic acid (CDG-3). Under Ar, a solution of lithium methoxide (10 mg, 0.26 mmol) in methanol (anhydrous, 0.3 mL) was added dropwise to a solution of **8** (62 mg, 0.065 mmol) in anhydrous THF (1 mL) at -78 ºC and the reaction was stirred for 5 min. *Tert*-butyl hypochlorite (15 μL, 0.13 mmol) was then added dropwise and the mixture was stirred at the same temperature for 30 min. Anhydrous THF (5 mL) was added to dilute and the resulting solution was poured in one port to an aqueous solution containing ammonium chloride and sodium bisulfite, extracted with ethyl acetate (15 mL x 3) and dried over $MgSO_4$. Compound **9** was obtained as crude product after solvent was removed, which was then treated with a mixture CH_2Cl_2/TFA/TIPS (3.8/0.2/0.05 mL) at 0 ºC for 3 h. The titled compound **CDG-3** (35.5 mg, 67% for 2 steps) was obtained after HPLC purification. ^{1}H NMR (400 MHz, d^6-DMSO) δ 9.55 (s, 1H), 7.46 (s, 1H), 7.41 (d, *J* = 8.5 Hz, 2H), 7.37 – 6.92 (m, 14H), 6.68 (d, *J* = 9.6 Hz, 1H), 6.57 (s, 1H), 5.29 (s, 1H), 5.25 (s, 2H), 4.44 (d, *J* = 11.3 Hz, 1H), 4.33 (d, *J* = 11.3 Hz, 1H), 3.84 (s, 3H), 3.59 (d, *J* = 14.1 Hz, 1H), 3.54 (d, *J* = 14.1 Hz, 1H), 3.35 (s, 3H), 1.98 (s, 3H), 1.49 – 1.43 (m, 1H), 1.39 – 1.33 (m, 1H), 1.26-1.20 (m, 1H), 0.94-0.89 (m, 1H); ^{13}C NMR (101 MHz, d^6-DMSO) δ 180.84, 172.41, 165.79, 163.58, 161.32, 160.94, 159.56, 158.70, 156.26, 138.17, 136.17, 132.36, 131.28, 131.04, 130.69, 129.84, 129.26, 128.92, 128.77, 127.22, 126.87, 125.86, 124.41, 118.39, 116.87, 116.57, 115.92, 115.22, 112.49, 104.54, 102.06, 96.04, 71.12, 64.66, 63.11, 55.97, 53.15, 42.31, 21.12, 20.22; HRMS: Calculated for $C_{47}H_{41}N_2O_{10}S^+$ ($[M+H]^+$): 825.2476; Found: 825.2469.

References

1. Wang F, Cassidy C, & Sacchettini JC (2006) Crystal structure and activity studies of the Mycobacterium tuberculosis beta-lactamase reveal its critical role in resistance to beta-lactam antibiotics. *Antimicrob. Agents Chemother.* 50(8):2762-2771.
2. Palmer KL, Aye LA, & Whiteley M (2007) Nutritional cues control Pseudomonas aeruginosa multicellular Behavior in cystic fibrosis sputum. *J. Bacteriol.* 189(22):8079-8087.
3. Hagmann WK*, et al.* (1989) Inhibition of Human-Leukocyte Elastase by C-2 Substituted Cephalosporin Sulfones. *E. J. Med. Chem.* 24(6):599-604.
4. Xie H*, et al.* (2012) Rapid point-of-care detection of the tuberculosis pathogen using a BlaC-specific fluorogenic probe. *Nat Chem* 4(10):802-809.
5. Pitlik J, Jaszberenyi JC, & Komaromi I (1991) Substituent Effects in the 1,3-Dipolar Cycloaddition Reactions of 2-Methylenecephalosporins with Diazoalkanes. *Liebigs Ann Chem* (7):699-701.
6. Belov VN, Wurm CA, Boyarskiy VP, Jakobs S, & Hell SW (2010) Rhodamines NN:

A Novel Class of Caged Fluorescent Dyes. *Angew Chem Int Edit* 49(20):3520-3523.
7. Koppel GA & Koehler RE (1973) Functionalization of C6(7) of Penicillins and Cephalosporins - One-Step Stereoselective Synthesis of 7-Alpha-Methoxycephalosporin C. *Journal of the American Chemical Society* 95(7):2403-2404.
8. Urano Y, *et al.* (2005) Evolution of fluorescein as a platform for finely tunable fluorescence probes. *J. Am. Chem. Soc.* 127(13):4888-4894.

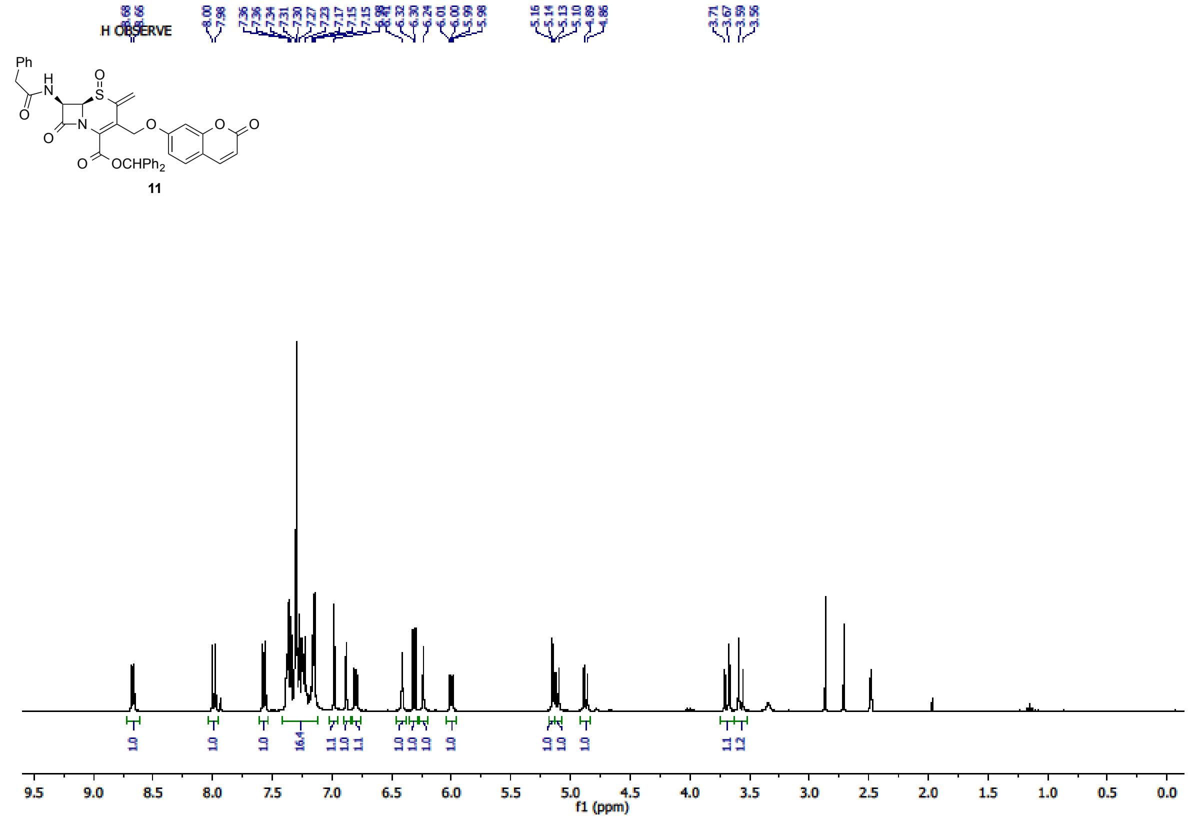
H OBSERVE
Ph
H
N
O
S
O
N
O
O
O
OCHPh2
O
O
O
1.0
1.0
1.0
16.4
1.1
1.0
1.1
1.0
1.0
1.0
1.0
1.0
1.0
1.0
1.1
1.2
f1 (ppm)

11

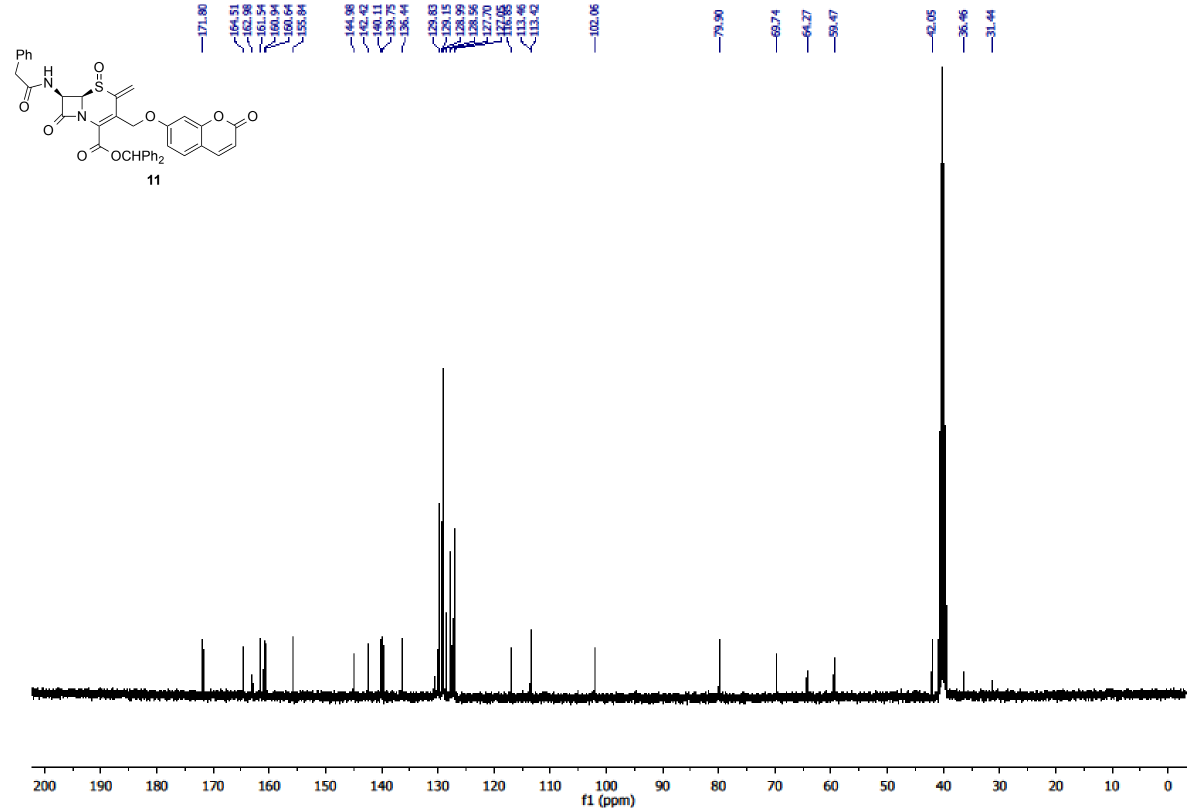
171.80
164.51
162.98
161.54
160.94
160.64
155.84
144.98
142.42
140.11
139.75
136.44
129.83
129.15
128.99
128.56
127.70
113.46
113.42
102.06
79.90
69.74
64.27
59.47
42.05
36.46
31.44
Ph
H
N
O
S
O
N
O
O
O
OCHPh2
O
O
O
f1 (ppm)

11

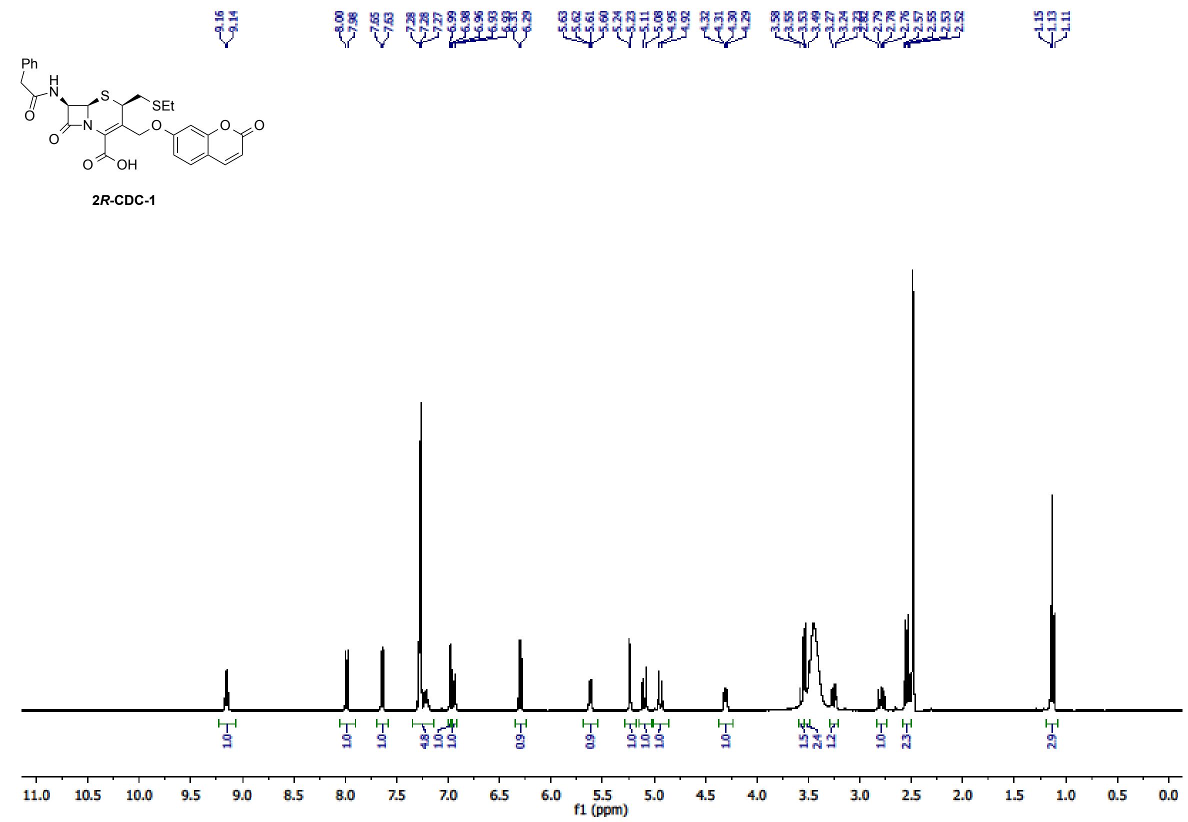
Ph
SEt
2R-CDC-1
f1 (ppm)

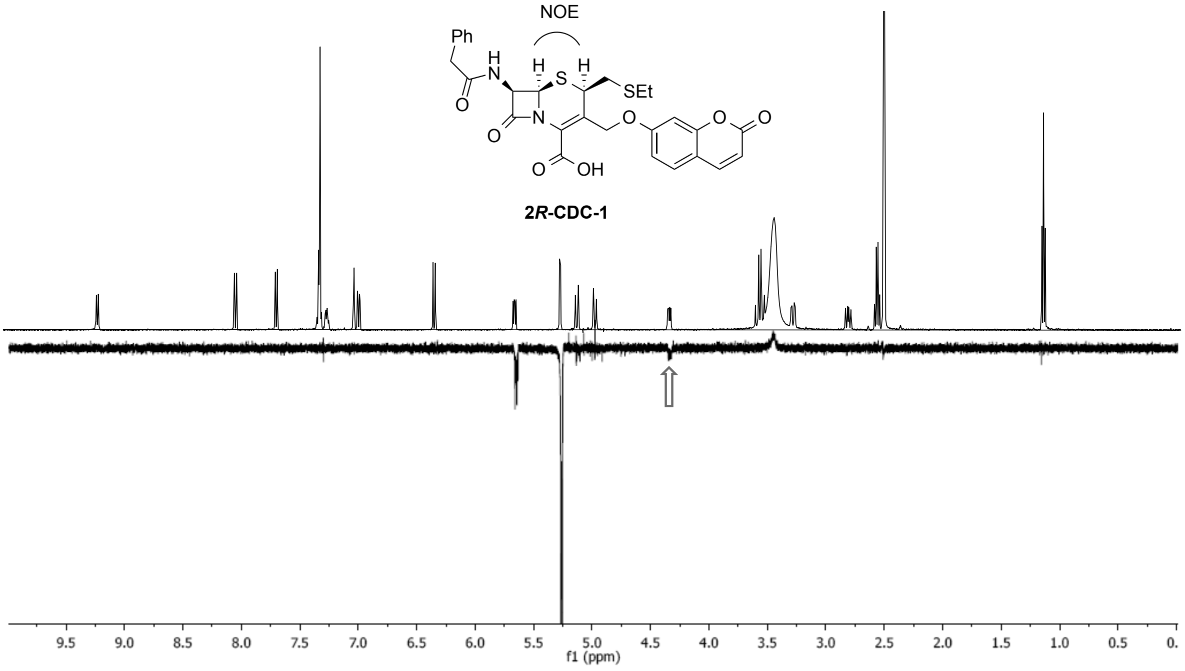
NOE
Ph
SEt
2R-CDC-1
f1 (ppm)

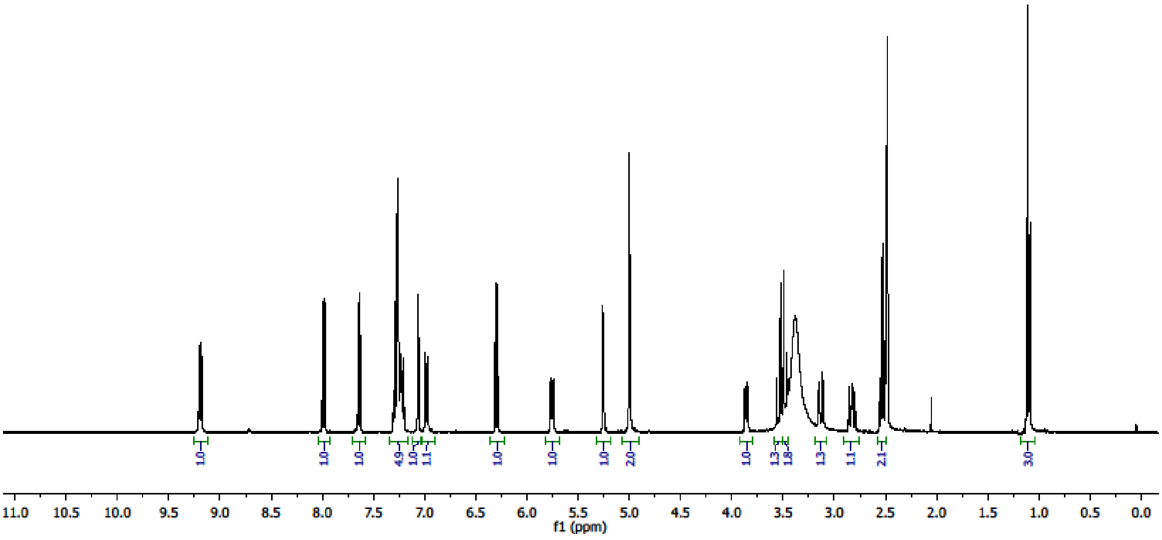
1.0
1.0
1.0
4.9
1.0
1.1
1.0
1.0
1.0
2.0
1.0
1.3
1.8
1.3
1.1
2.1
3.0
11.0 10.5 10.0 9.5 9.0 8.5 8.0 7.5 7.0 6.5 6.0 5.5 5.0 4.5 4.0 3.5 3.0 2.5 2.0 1.5 1.0 0.5 0.0
f1 (ppm)

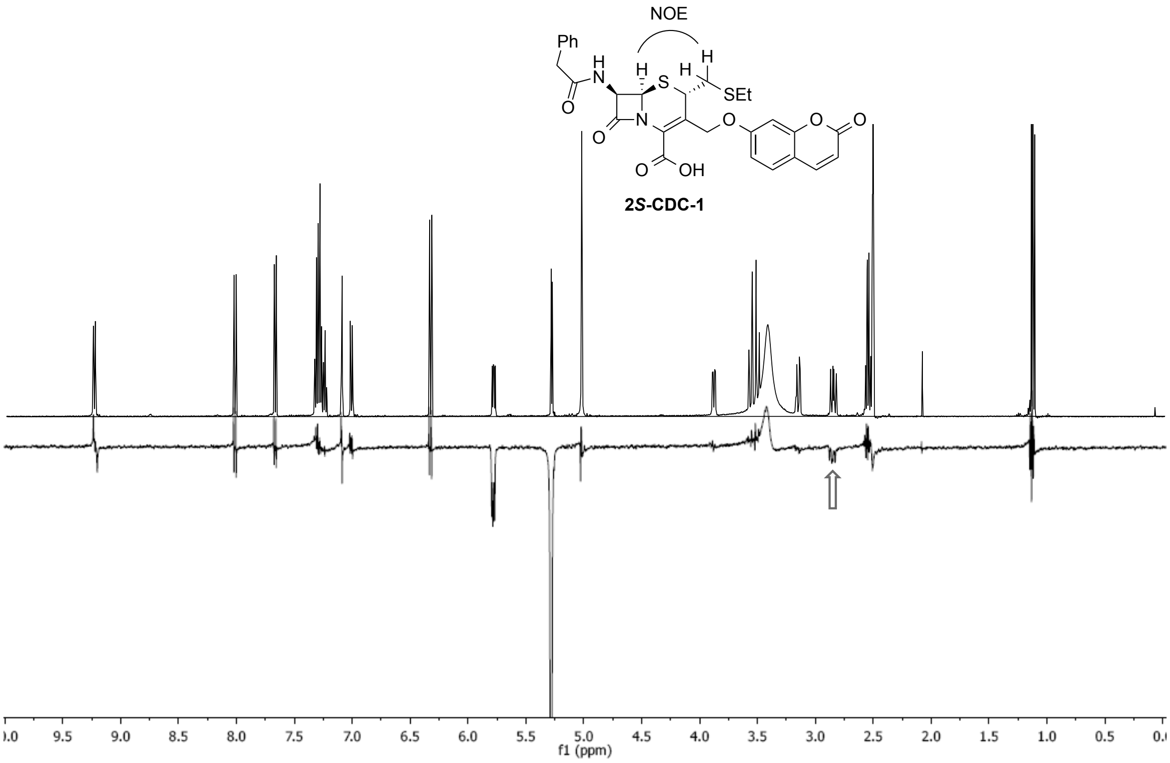
NOE
Ph
H
N
H
S
H
H
SEt
O
N
O
O
O
O
O
OH
2S-CDC-1
9.5 9.0 8.5 8.0 7.5 7.0 6.5 6.0 5.5 5.0 4.5 4.0 3.5 3.0 2.5 2.0 1.5 1.0 0.5
f1 (ppm)

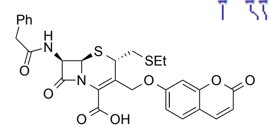
Ph
H
N
S
SEt
O
OH

2S-CDC-1

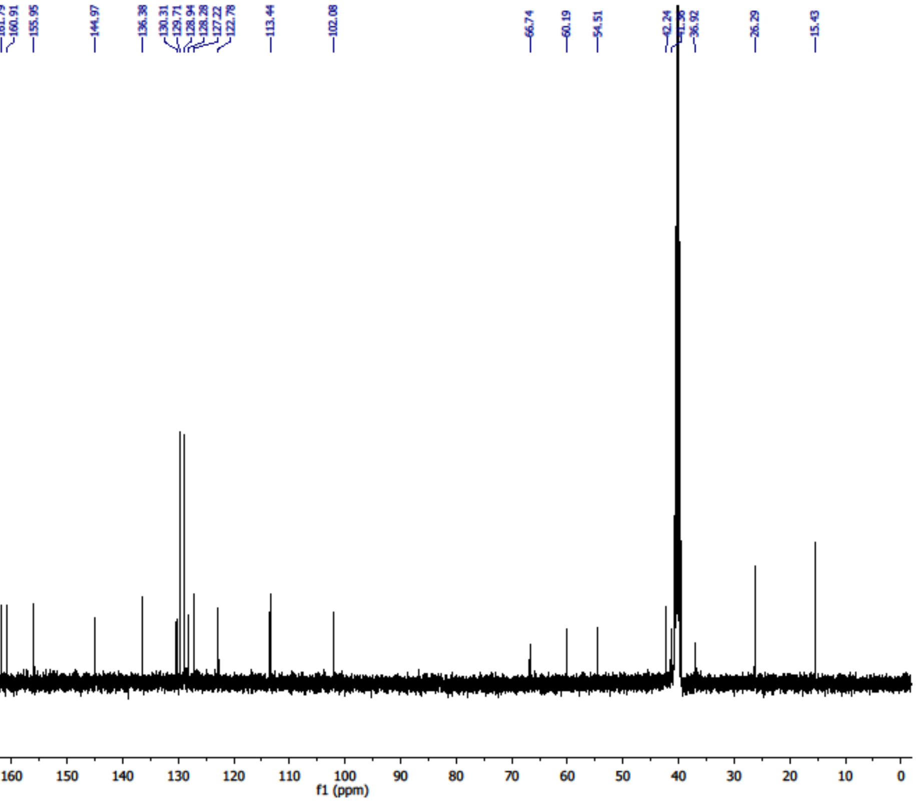
171.62
165.30
163.71
161.79
160.91
155.95
144.97
136.38
130.31
129.71
128.94
128.28
127.22
122.78
113.44
102.08
66.74
60.19
54.51
42.24
36.92
26.29
15.43
200 190 180 170 160 150 140 130 120 110 100 90 80 70 60 50 40 30 20 10 0
f1 (ppm)

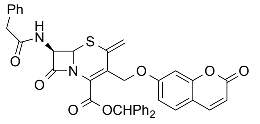
Ph
H
N
S
O
OCHPh2

14

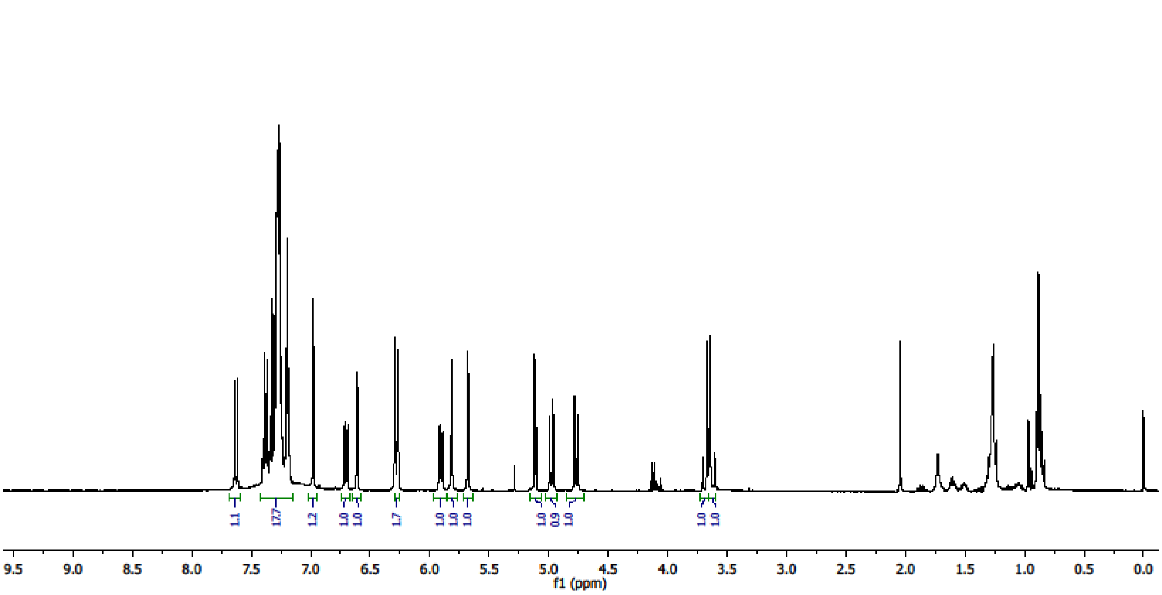
7.61
7.40
7.38
7.37
7.34
7.32
7.30
7.28
7.28
7.26
7.25
7.25
7.23
7.20
7.20
7.19
7.18
7.17
6.98
6.71
6.69
6.61
6.61
6.29
6.26
5.92
5.91
5.90
5.89
5.81
5.68
5.12
5.11
4.99
4.96
4.78
4.76
3.70
3.66
3.64
3.60
1.1
17.7
1.2
1.0
1.0
1.7
1.0
1.0
1.0
1.0
0.9
1.0
1.0
1.0
9.5 9.0 8.5 8.0 7.5 7.0 6.5 6.0 5.5 5.0 4.5 4.0 3.5 3.0 2.5 2.0 1.5 1.0 0.5 0.0
f1 (ppm)

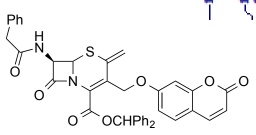

14

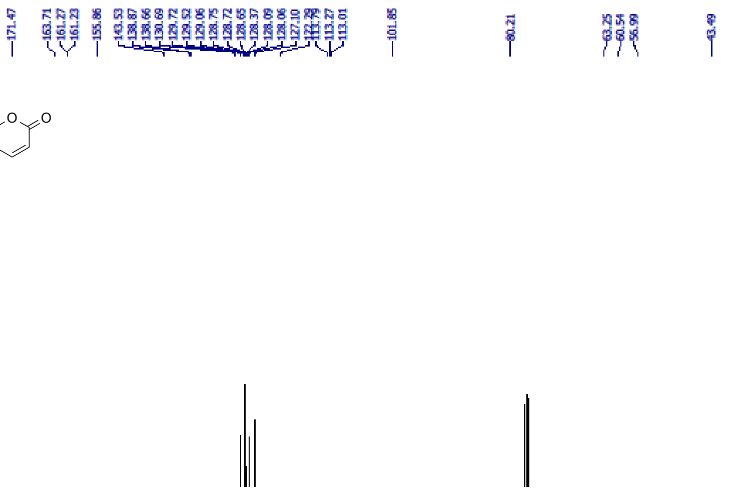

171.47
163.71
161.27
161.23
155.86
143.53
138.87
138.66
130.69
129.72
129.52
129.06
128.75
128.72
128.65
128.37
128.09
128.06
127.10
113.27
113.01
101.85
80.21
63.25
60.54
56.99
43.49

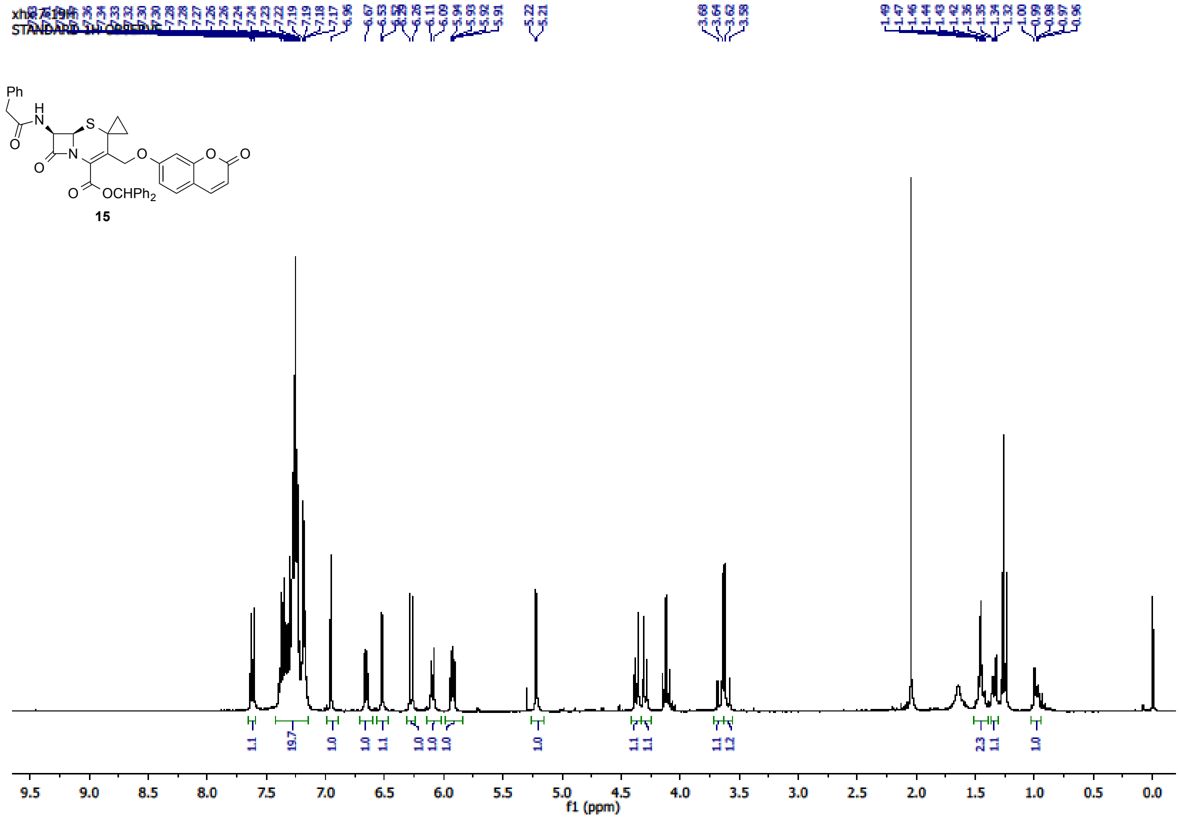

6.96
6.67
6.53
6.25
6.11
6.09
5.94
5.93
5.92
5.91
5.22
5.21
3.68
3.64
3.62
3.58
1.49
1.47
1.46
1.44
1.43
1.42
1.36
1.35
1.34
1.32
1.00
0.99
0.98
0.97
0.96
1.1
19.7
1.0
1.0
1.1
1.0
1.0
1.0
1.0
1.1
1.1
1.1
1.2
2.3
1.1
1.0
9.5 9.0 8.5 8.0 7.5 7.0 6.5 6.0 5.5 5.0 4.5 4.0 3.5 3.0 2.5 2.0 1.5 1.0 0.5 0.0
f1 (ppm)

15

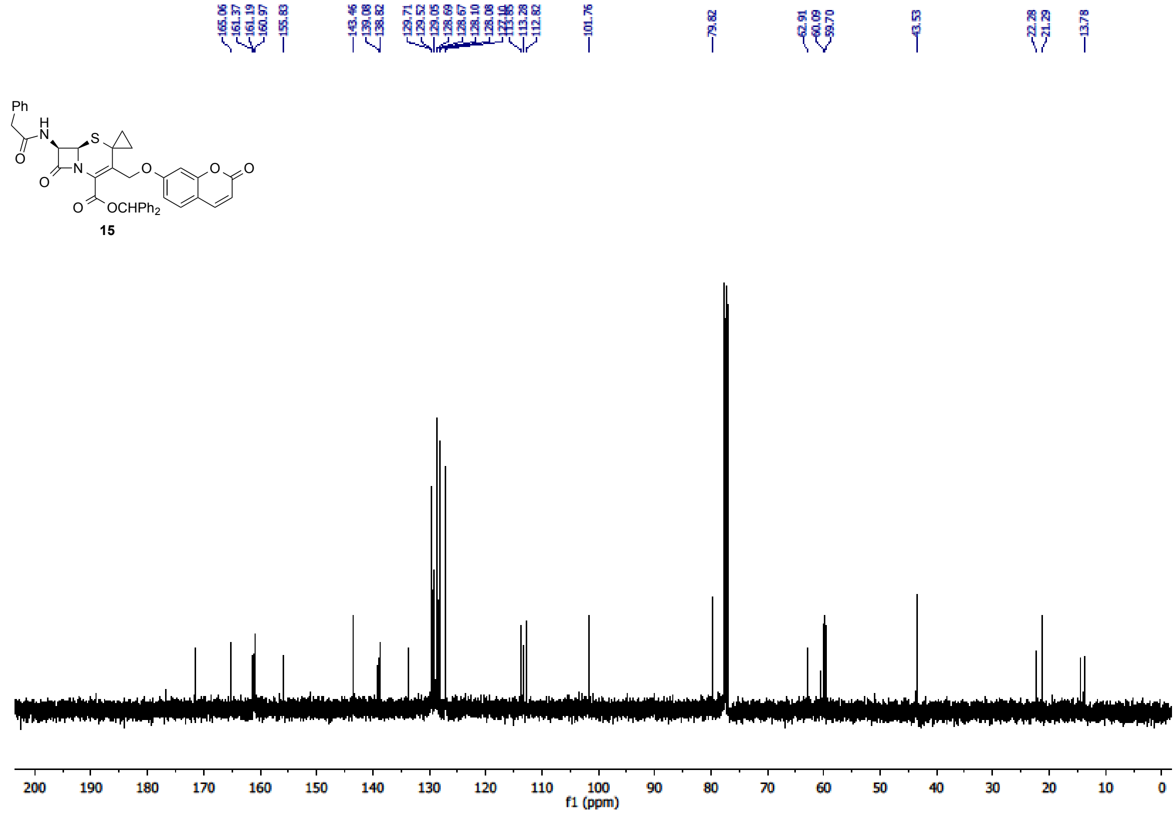
15
f1 (ppm)

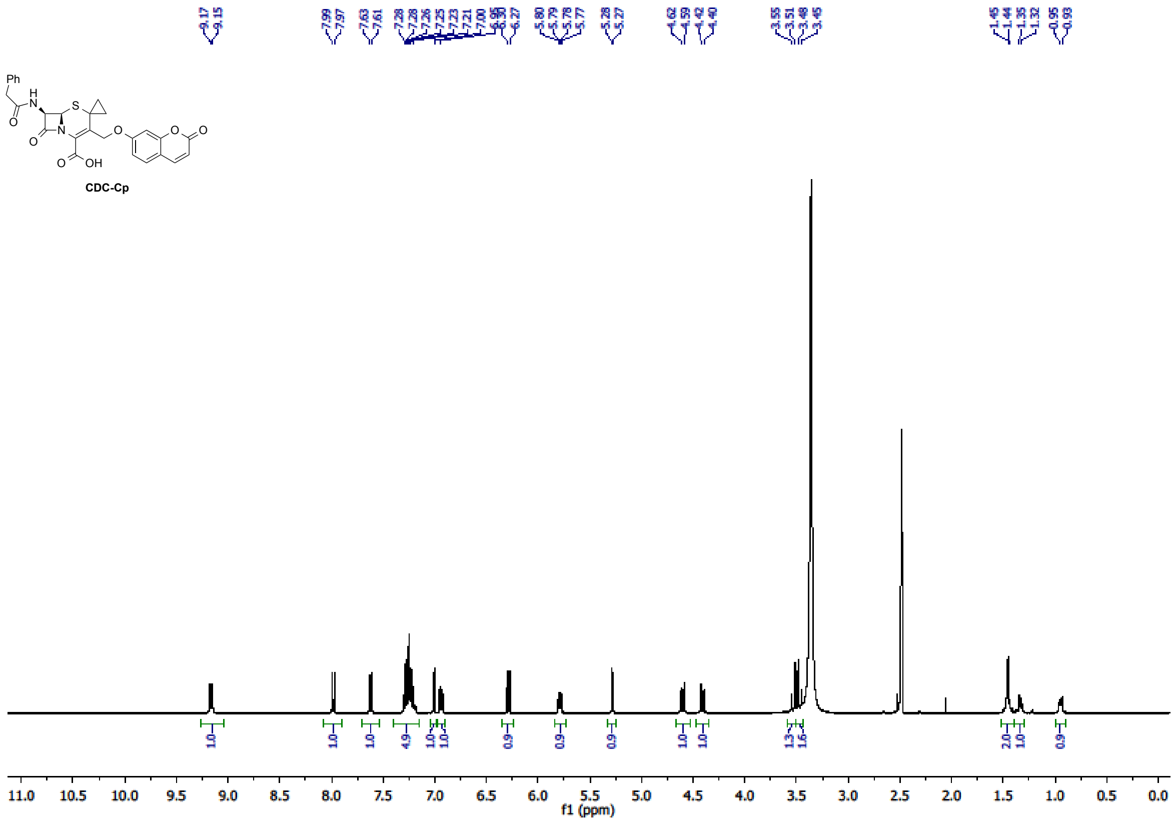
CDC-Cp
f1 (ppm)

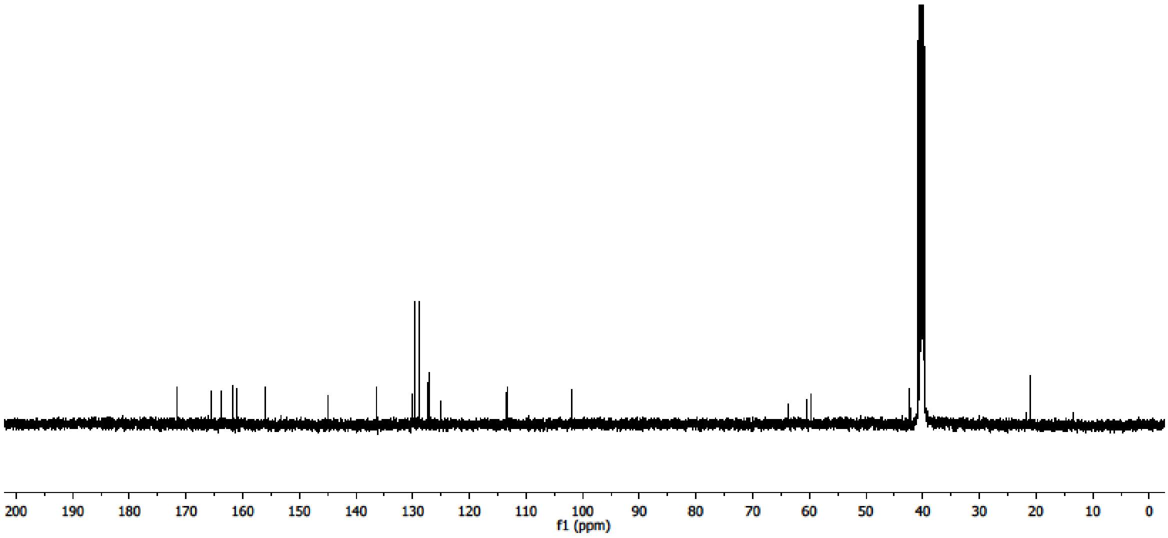
f1 (ppm)

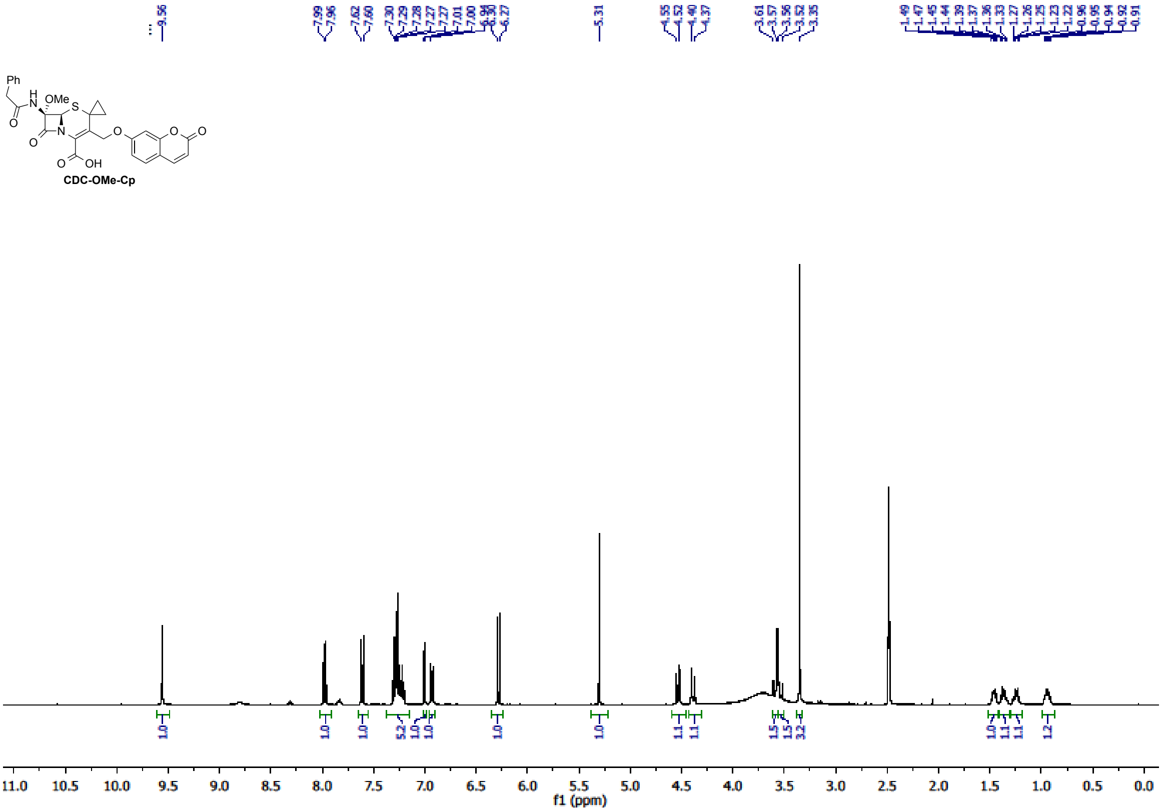
CDC-OMe-Cp
f1 (ppm)

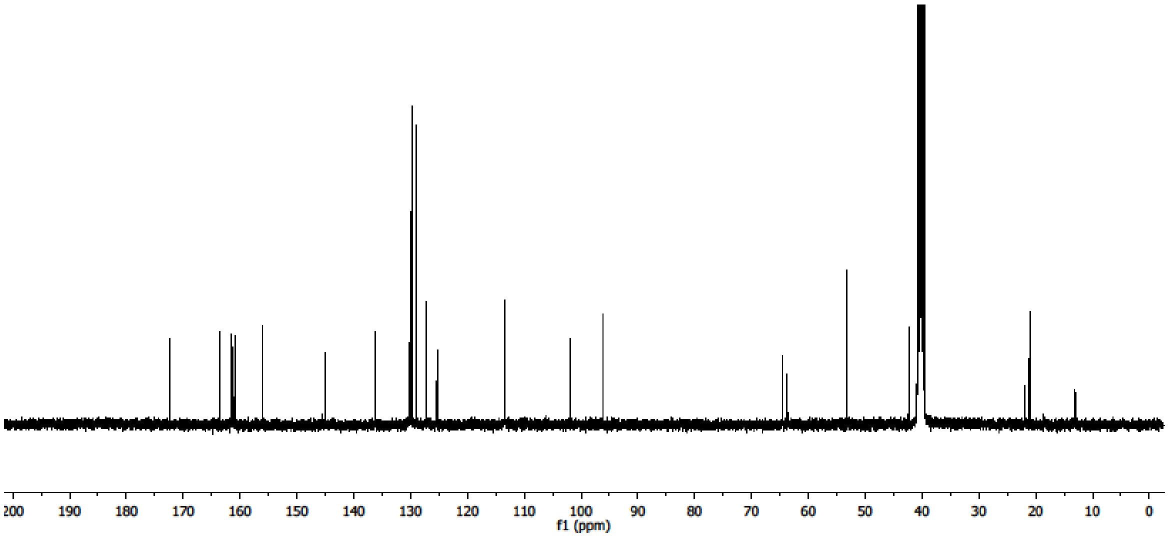

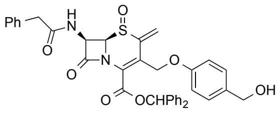

4

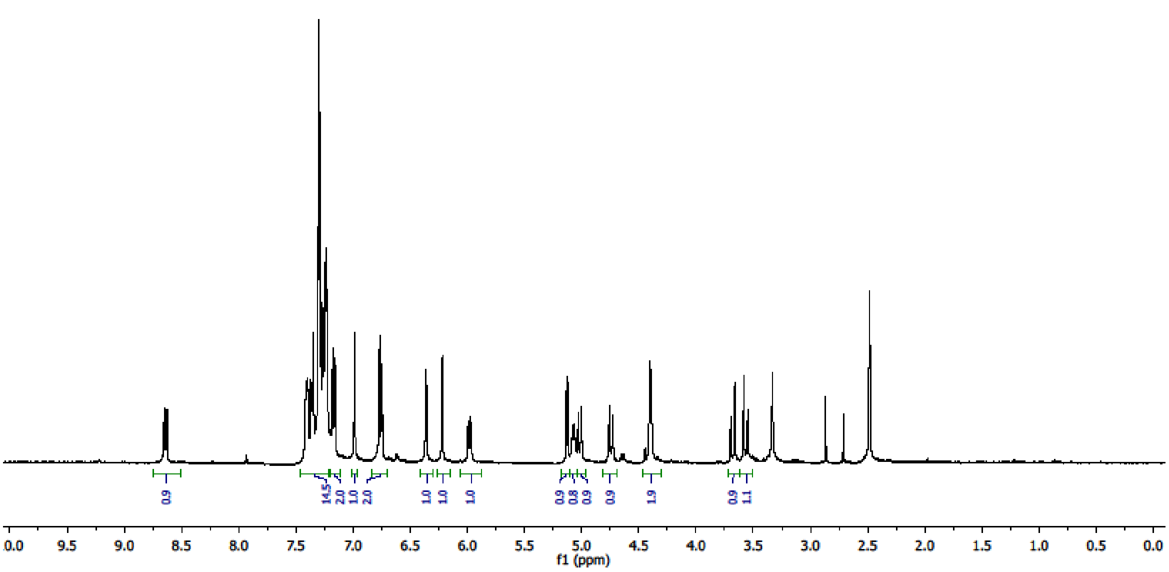

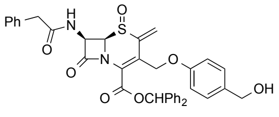
Ph
H
N
O
S
O
N
O
O
OCHPh$_2$
OH

4

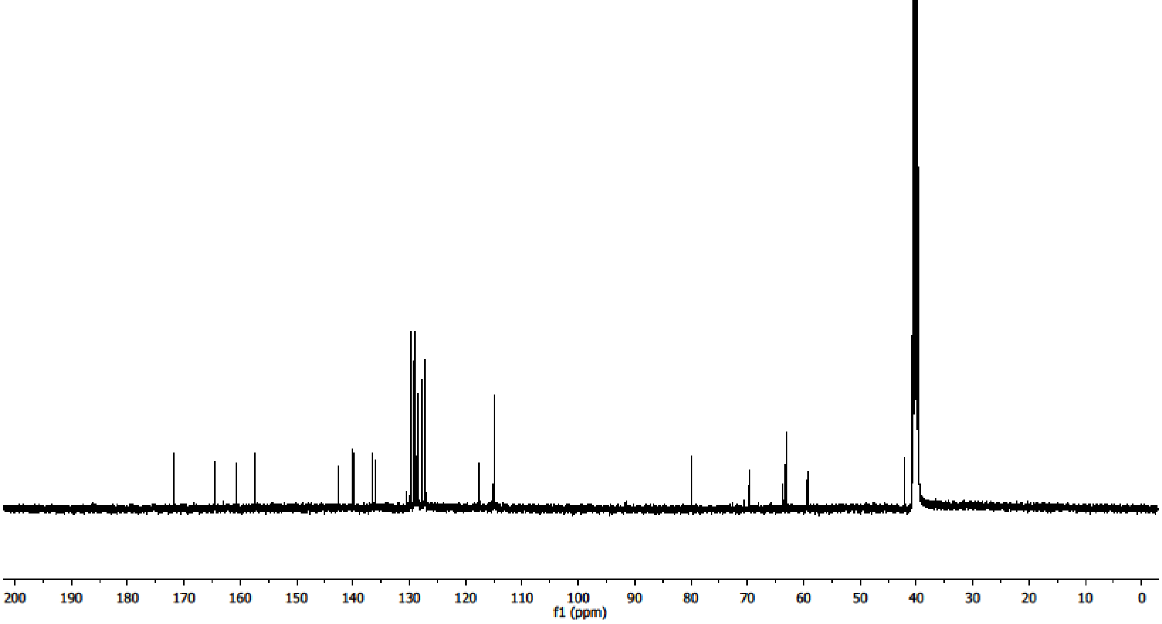
200 190 180 170 160 150 140 130 120 110 100 90 80 70 60 50 40 30 20 10 0
f1 (ppm)

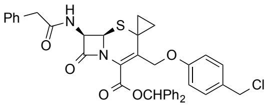
Ph
H
N
O
S
O
N
O
O
OCHPh$_2$
Cl

7

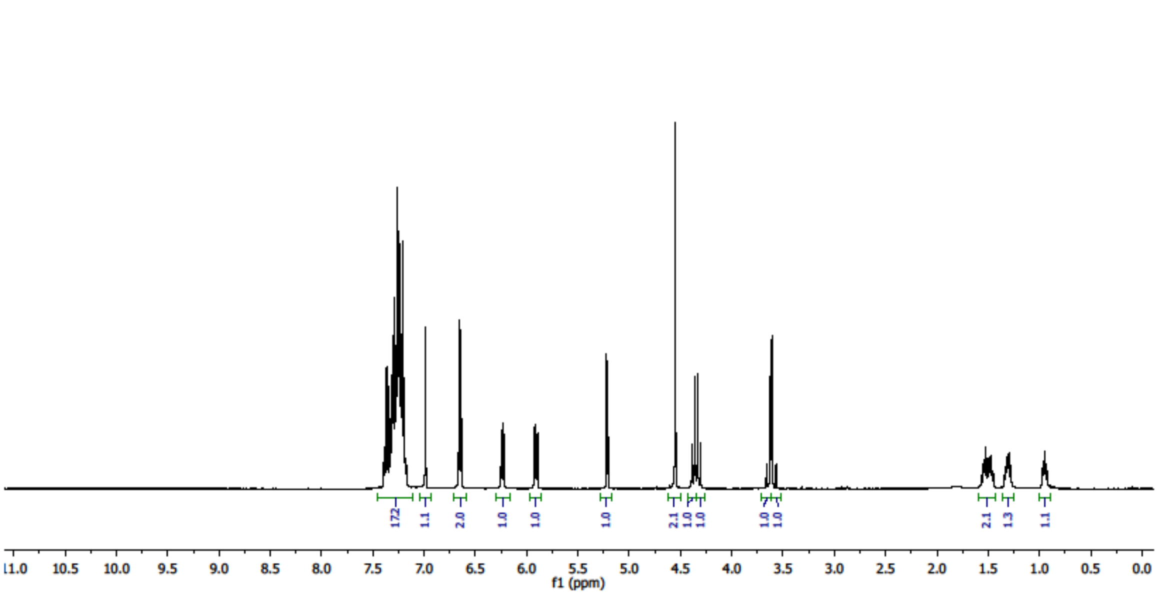
17.2
1.1
2.0
1.0
1.0
1.0
2.1
1.0
1.0
1.0
1.0
2.1
1.3
1.1
11.0 10.5 10.0 9.5 9.0 8.5 8.0 7.5 7.0 6.5 6.0 5.5 5.0 4.5 4.0 3.5 3.0 2.5 2.0 1.5 1.0 0.5 0.0
f1 (ppm)

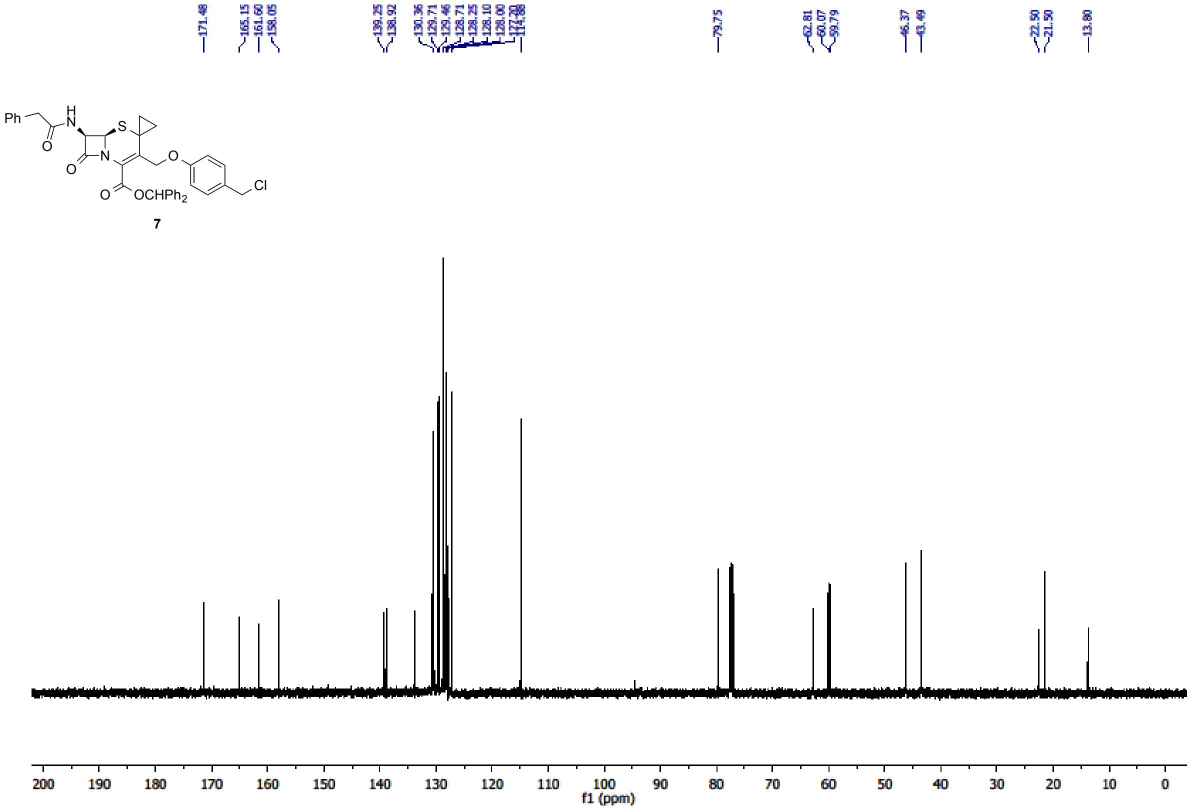

171.48
165.15
161.60
158.05
139.25
138.92
130.36
129.71
129.46
128.71
128.25
128.10
128.00
114.88
79.75
62.81
60.07
59.79
46.37
43.49
22.50
21.50
13.80
Ph
H
N
S
O
N
O
O
Cl
O
OCHPh2
f1 (ppm)

7

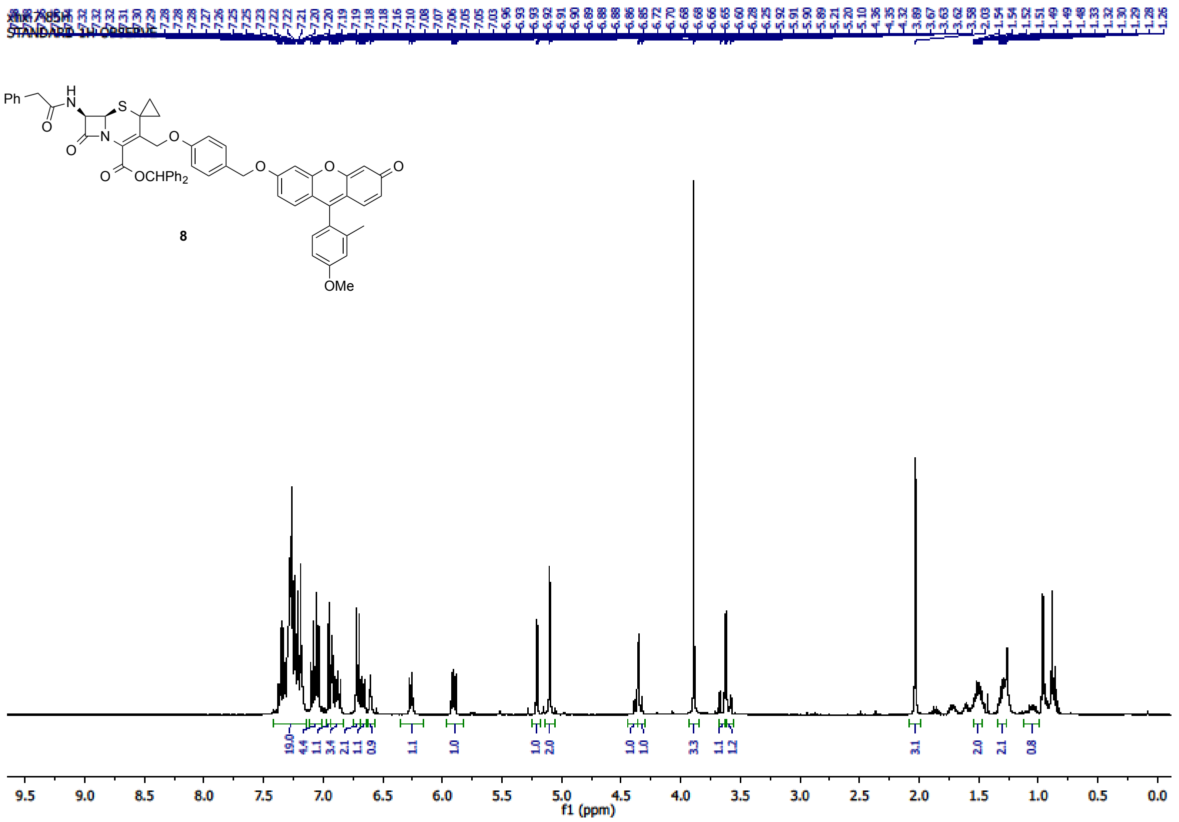

Ph
H
N
S
O
N
O
O
O
O
O
OCHPh2
OMe
19.0
4.4
1.1
3.4
2.1
1.1
0.9
1.1
1.0
1.0
2.0
1.0
1.0
3.3
1.1
1.2
3.1
2.0
2.1
0.8
f1 (ppm)

8

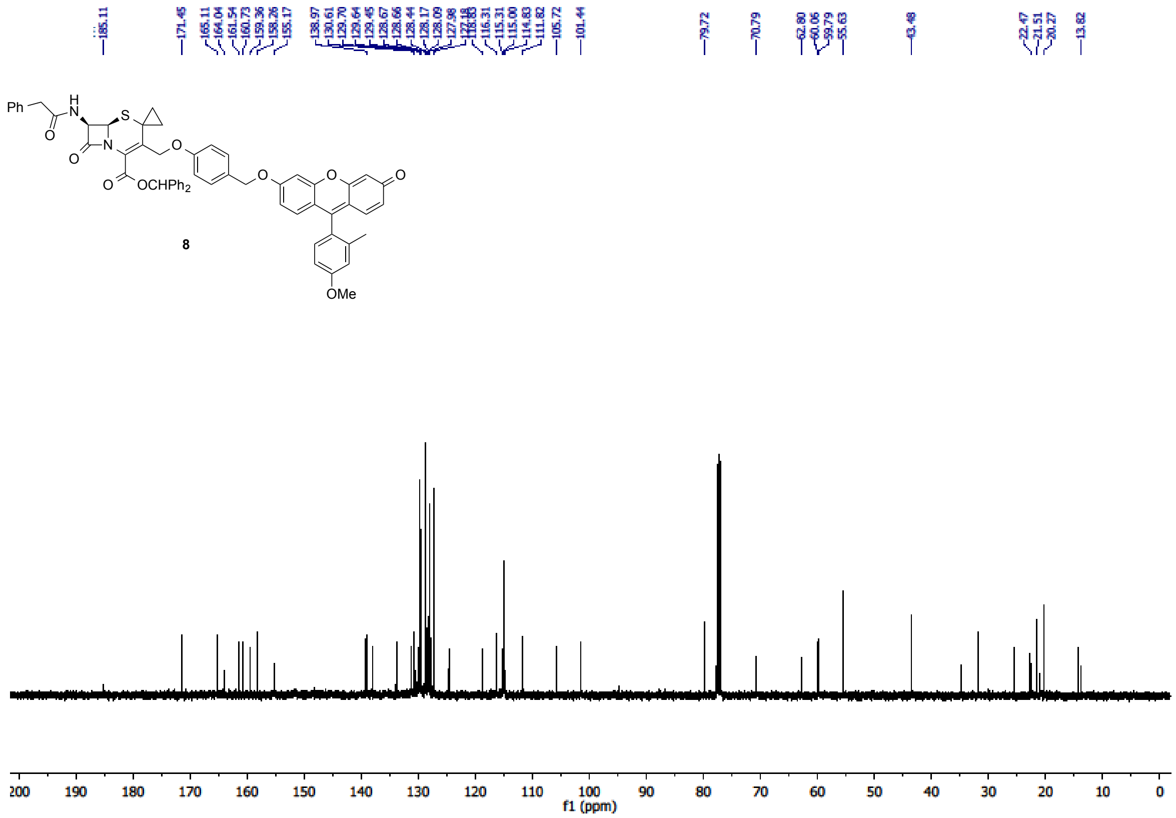
8
f1 (ppm)

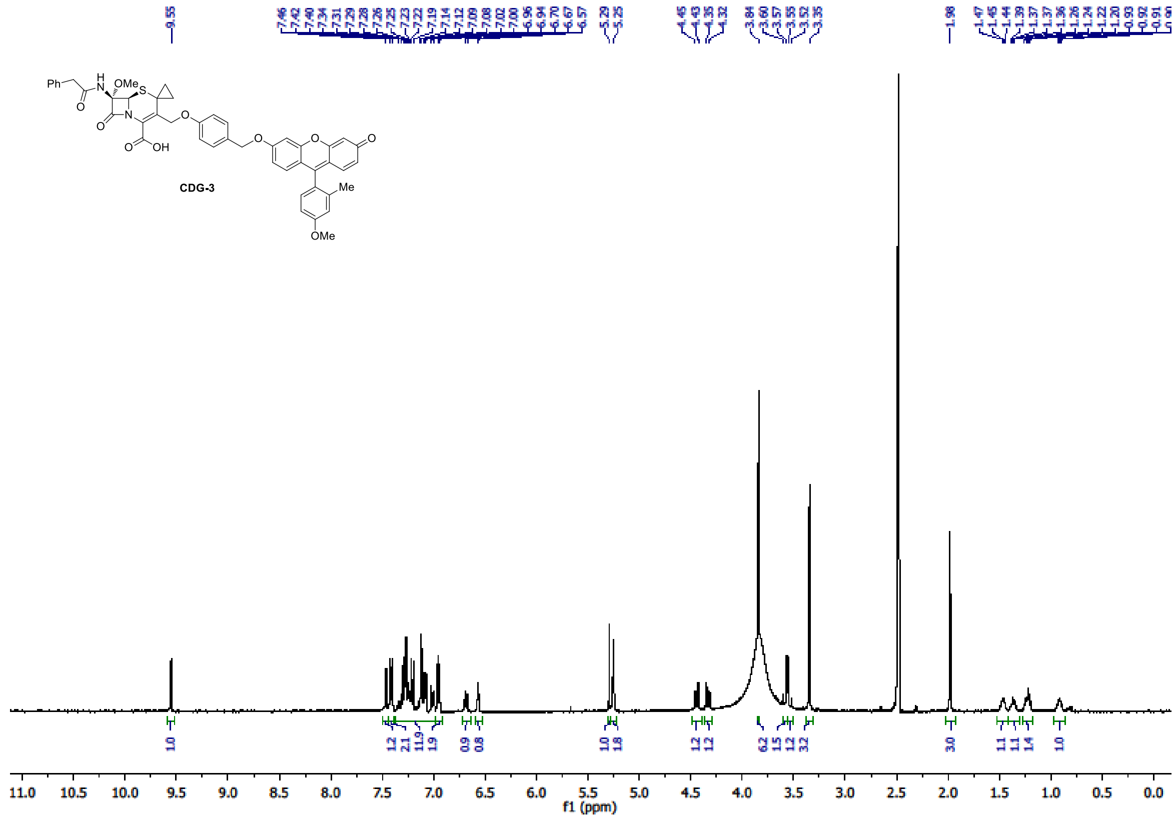
CDG-3
f1 (ppm)

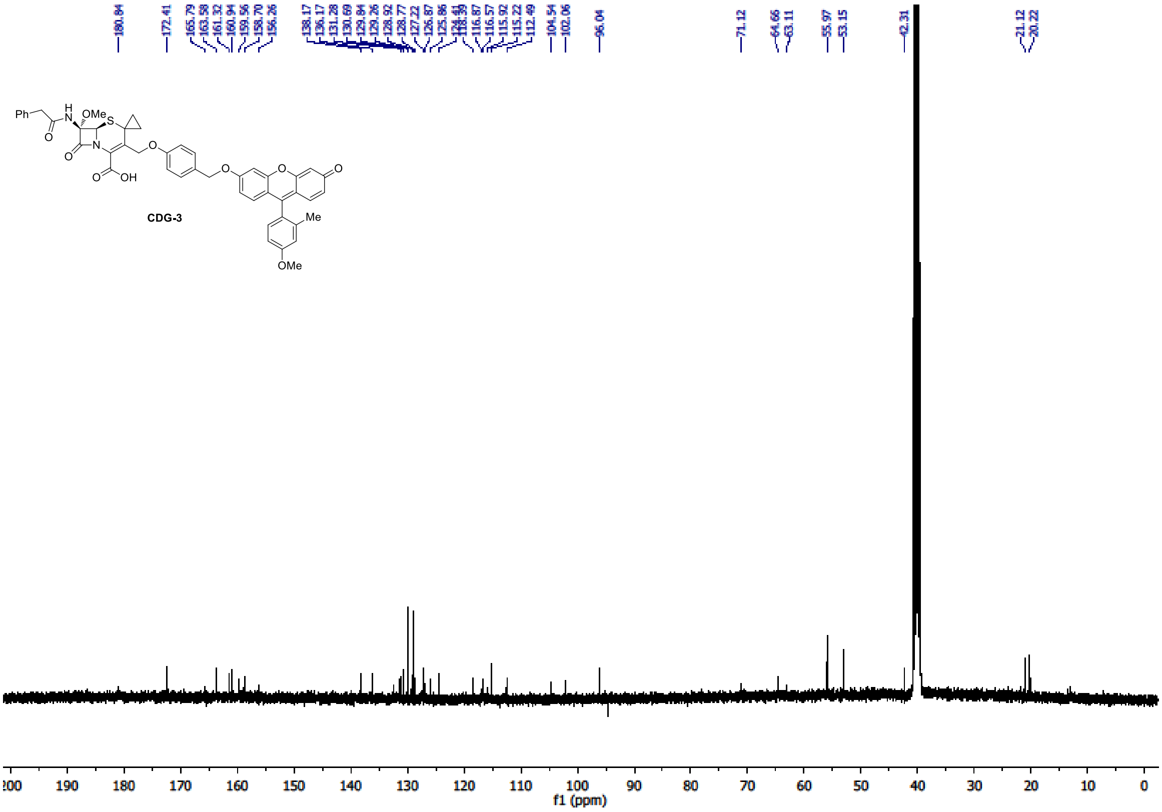
180.84
172.41
165.79
163.58
161.32
160.94
159.56
158.70
156.26
138.17
136.17
131.28
130.69
129.84
129.26
128.92
128.77
127.22
126.87
125.86
116.87
116.57
115.92
115.22
112.49
104.54
102.06
96.04
71.12
64.66
63.11
55.97
53.15
42.31
21.12
20.22
CDG-3
f1 (ppm)
